# Schnyder corneal dystrophy-associated UBIAD1 mutations cause corneal cholesterol accumulation by stabilizing HMG-CoA reductase

**DOI:** 10.1371/journal.pgen.1008289

**Published:** 2019-07-19

**Authors:** Shi-You Jiang, Jing-Jie Tang, Xu Xiao, Wei Qi, Suqian Wu, Chao Jiang, Jiaxu Hong, Jianjiang Xu, Bao-Liang Song, Jie Luo

**Affiliations:** 1 Hubei Key Laboratory of Cell Homeostasis, College of Life Sciences, Wuhan University, Wuhan, China; 2 The State Key Laboratory of Molecular Biology, Institute of Biochemistry and Cell Biology, University of Chinese Academy of Sciences, Chinese Academy of Sciences, Shanghai, China; 3 School of Life Science and Technology, ShanghaiTech University, Shanghai, China; 4 Department of Ophthalmology and Visual Science, Eye Institute, Eye and ENT Hospital, Shanghai Medical College of Fudan University, NHC Key Laboratory of myopia (Fudan University), Shanghai Key Laboratory of Visual Impairment and Restoration, Shanghai, China; 5 Shenzhen Institute of Wuhan University, Shenzhen, China; University of Michigan, UNITED STATES

## Abstract

Schnyder corneal dystrophy (SCD) is a rare genetic eye disease characterized by corneal opacification resulted from deposition of excess free cholesterol. UbiA prenyltransferase domain-containing protein-1 (UBIAD1) is an enzyme catalyzing biosynthesis of coenzyme Q10 and vitamin K_2_. More than 20 UBIAD1 mutations have been found to associate with human SCD. How these mutants contribute to SCD development is not fully understood. Here, we identified HMGCR as a binding partner of UBIAD1 using mass spectrometry. In contrast to the Golgi localization of wild-type UBIAD1, SCD-associated mutants mainly resided in the endoplasmic reticulum (ER) and competed with Insig-1 for HMGCR binding, thereby preventing HMGCR from degradation and increasing cholesterol biosynthesis. The heterozygous *Ubiad1* G184R knock-in (*Ubiad1*^*G184R/+*^) mice expressed elevated levels of HMGCR protein in various tissues. The aged *Ubiad1*^*G184R/+*^ mice exhibited corneal opacification and free cholesterol accumulation, phenocopying clinical manifestations of SCD patients. In summary, these results demonstrate that SCD-associated mutations of UBIAD1 impair its ER-to-Golgi transportation and enhance its interaction with HMGCR. The stabilization of HMGCR by UBIAD1 increases cholesterol biosynthesis and eventually causes cholesterol accumulation in the cornea.

## Author summary

Schnyder corneal dystrophy (SCD) is a rare genetic eye disease caused by deposition of free cholesterol in the cornea. It is closely correlated with mutations in the UbiA prenyltransferase domain-containing protein-1 (*UBIAD1*) gene, which encodes an enzyme catalyzing biosynthesis of coenzyme Q10 and vitamin K_2_. The underlying mechanism by which UBIAD1 mutations result in SCD development is unclear. Here, we report that SCD-associated mutations trap UBIAD1 in the ER and block Insig-1 mediated HMGCR degradation. We also generated a heterozygous mouse model (*Ubiad1*^*G184R/+*^) that mimics human SCD. We conclude that SCD-associated UBIAD1 mutations decrease HMGCR degradation and subsequently increase cholesterol biosynthesis in the cornea.

## Introduction

Schnyder corneal dystrophy (SCD) is a rare autosomal dominant genetic eye disease [1]. It is characterized by free cholesterol accumulation in the cornea that causes progressive corneal opacification with aging [1] [2]. Genetics studies have linked SCD to mutations in UbiA prenyltransferase domain-containing protein-1 (*UBIAD1*), also known as transitional epithelial response gene 1 (*TERE1*) [3–5]. Until now, 25 missense mutations altering 21 amino acids, including N102S and G186R (equivalent to N100S and G184R in mouse *Ubiad1*, respectively), have been identified in about 50 SCD families [6–8]. However, the causal relationship between UBIAD1 mutations and SCD development has not been proved until very recently [9].

The UBIAD1 protein belongs to the UbiA superfamily of intramembrane aromatic prenyltransferases, which catalyze the biosynthesis of a variety of lipophilic molecules such as ubiquinones, vitamin K, vitamin E, chlorophylls, hemes and archaeal tetraether lipids [10]. UBIAD1 has been identified as a vitamin K_2_ biosynthesis enzyme in humans and mice, and is essential for mouse embryonic development [11, 12]. In Drosophila, the UBIAD1 homolog was reported to generate vitamin K_2_ that functions as an electron carrier for sustaining mitochondrial function [13]. In zebrafish, UBIAD1 was proposed to synthesize ubiquinone coenzyme Q10 that protects against oxidative damages through regulating nitric oxide activity, thereby maintaining vascular endothelial cell survival [14]. However, how UBIAD1 mutations cause cholesterol accumulation in the cornea is not fully understood.

The endoplasmic reticulum (ER)-localized 3-hydroxy-3-methyglutaryl coenzyme A reductase (HMG-CoA reductase, HMGCR) is a rate-limiting enzyme of the cholesterol biosynthetic pathway catalyzing the synthesis of mevalonate [15]. As an important intermediate, mevalonate gives rise to not only cholesterol, but also nonsterol isoprenoids such as farnesyl pyrophosphate and geranylgeranyl pyrophosphate (GGPP) that further generates ubiquinone, dolichol and hemes [15]. Interestingly, these mevalonate-derived molecules are also the final products of UBIAD1 superfamily members, suggesting a potential link between HMGCR and UBIAD1. Theses sterols and nonsterol isoprenoids coordinate to accelerate degradation of HMGCR to prevent over-accumulation of cholesterol [16]. Accumulating sterols induce HMGCR binding to ER-anchored Insig-1 and Insig-2, which bring ubiquitin ligases gp78, TRC8 and RNF145 together with other cofactors to ubiquitinate HMGCR, resulting in its degradation in proteasome [17–22]. Elucidating the molecular pathway of HMGCR degradation is of potential clinic significance. Statins as competitive inhibitors of HMGCR can decrease the synthesis of sterols and nonsterol isoprenoids and dramatically increase HMGCR in the liver [23, 24]. The lanosterol analog HMG499 (also named Cmpd 81) can potentiate the cholesterol-lowering effect of statins through inducing HMGCR degradation [25].

Recently, Ubiad1 was found to be a HMGCR-binding protein through proximity-dependent biotinylation using HMGCR as the bait [26]. The homozygous *Ubiad1* N100S knock-in (*Ubiad1*^*N100S/N100S*^) mice exhibit corneal opacification and HMGCR accumulation in tissues [9]. In this study, we identified HMGCR as a UBIAD1-associated protein through UBIAD1 immunoprecipitation coupled with mass spectrometry. We demonstrated that UBIAD1 competed with Insig-1 for binding HMGCR, preventing the latter from ubiquitination and degradation. All known SCD-associated UBIAD1 mutants localized in the ER and stabilized HMGCR protein. More importantly, we generated a different SCD-associated knock-in mouse line carrying *Ubiad1* G184R mutation. The *Ubiad1*^*G184R/+*^ mice exhibited excess HMGCR protein in tissues and striking corneal opacifications with free cholesterol deposition. These phenotypes recapitulate clinical manifestations of human SCD patients, suggesting that *Ubiad1*^*G184R/+*^ mouse is an ideal model to study human SCD.

## Results

### SCD-associated G186R UBIAD1 blocks degradation of HMGCR

To explore the underlying connections between UBIAD1 and cholesterol metabolism, we performed a tandem affinity purification (TAP) coupled to mass spectrometry to identify UBIAD1-associated proteins, using HEK-293 cells stably expressing human wild-type (WT) or G186R mutant form of UBIAD1 fused with a TAP tag at the C terminus (S1A Fig). The G186R mutation is a mutation found in an early-onset SCD family [27]. Besides, according to the determined structure of archaeal UbiA, the G186 residue was proposed to locate in the surface-exposed loop, and the G186R mutation may affect the interactions with other proteins [10, 28]. We found three proteins among the top of the list: HMGCR, VCP/p97 and SEL1L (S1B and S1C Fig, S1 Table). HMGCR is the rate-limiting enzyme converting HMG-CoA to mevalonate in the cholesterol biosynthetic pathway [15]. VCP/p97 and SEL1L have been known to be involved in the degradation of HMGCR and other ER proteins [29–31].

**Fig 1:**
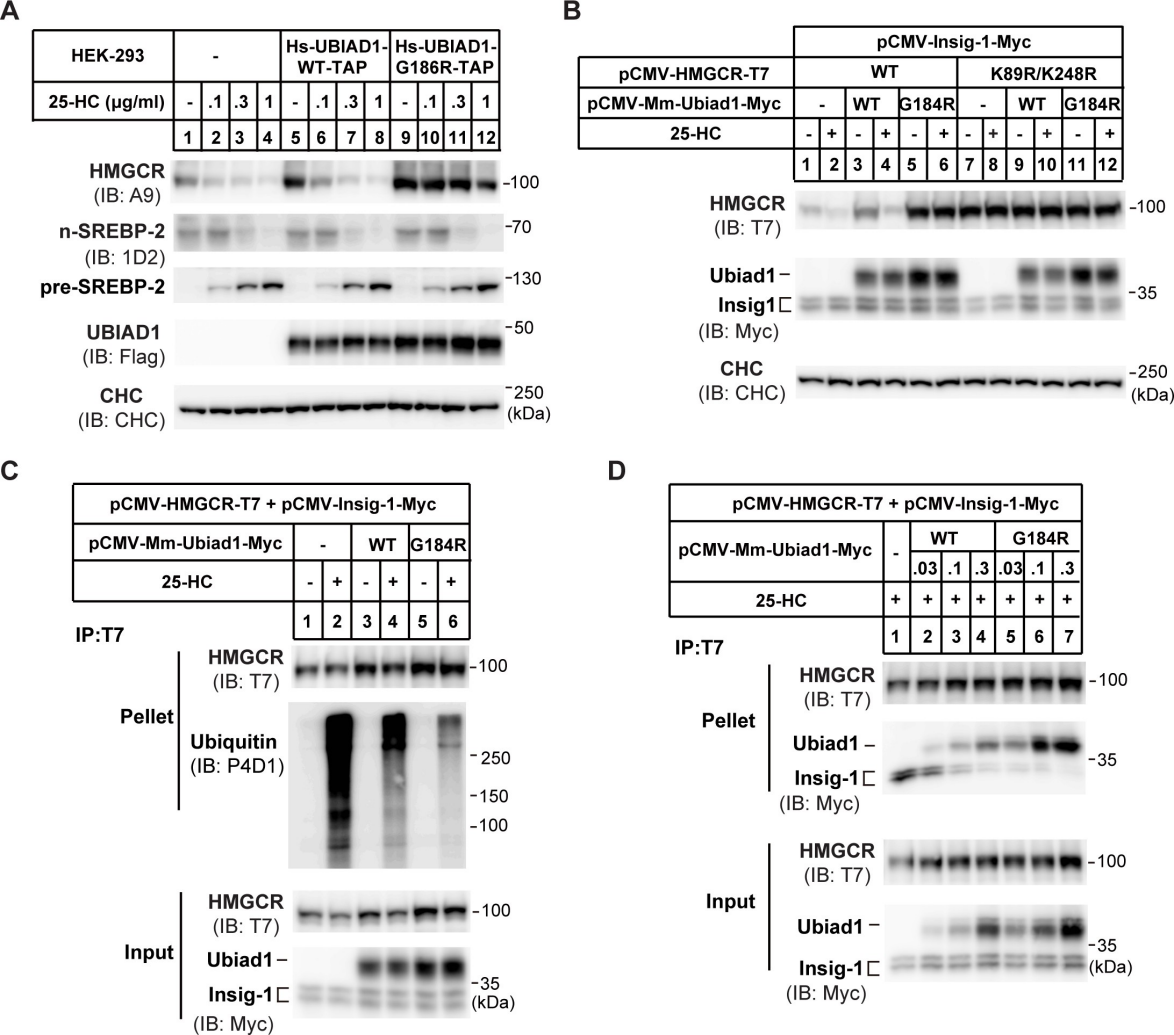
UBIAD1 (G186R) blocks the ubiquitination and degradation of HMGCR. (A) Effects of human wild-type (WT) UBIAD1 and mutated UBIAD1 (G186R) (equivalent to mouse G184R) on HMGCR protein level. HEK-293 cells stably expressing human (homo sapiens, Hs) WT UBIAD1-TAP and UBIAD1 (G186R)-TAP, respectively, were depleted of sterols in sterol-depleted medium containing 10% LPDS, 1 μM lovastatin and 50 μM mevalonate for 16 hr. Then indicated concentrations of 25-hydroxylcholesterol (25-HC) were added and incubated for 5 hr. Cells were harvested and subjected to immunoblot with antibodies against HMGCR (A9), SREBP-2 (1D2), and UBIAD1 (anti-Flag). Clathrin heavy chain (CHC) was a loading control. TAP: Flag-Protein A tags for tandem affinity purification in S1 Fig. (B) Effects of mouse WT and G184R Ubiad1 on the degradation of the ubiquitin sites mutated (K89R/K248R) HMGCR. CHO-K1 cells were transfected with indicated plasmids, depleted of sterols for 16 hr and treated with 1 μg/ml 25-HC and 10 mM mevalonate (Mev) for 5 hr. Lysates were immunoblotted with indicated antibodies. Mus musculus, Mm. (C) Effects of WT and G184R Ubiad1 on the ubiquitination of HMGCR. CHO-K1 cells were transfected and depleted of sterols, then cells were treated with1 μg/ml 25-HC, 10 mM mevalonate and 20 μM MG-132 for 2 hr before immunoprecipitation. Lysates were immunoprecipitated with anti-T7 antibody coupled agarose, and pellets and inputs were probed for indicated proteins. (D) Interaction between Ubiad1 and HMGCR. CHO-K1 cells were transfected, depleted of sterols and treated with 25-HC for 1 hr, then lysates were immunoprecipitated with anti-T7 antibody coupled agarose. Pellets and inputs were probed with indicated antibodies. The experiments are repeated three times and representative data are shown.

Therefore, we focused on the effects of UBIAD1 on sterol-induced degradation of HMGCR protein. The cells stably expressing WT UBIAD1 or G186R mutant were treated with increasing concentrations of 25-hydroxycholesterol (25-HC) for 5 h. The endogenous HMGCR was degraded in a concentration-dependent manner in cells stably expressing UBIAD1 (WT). However, 25-HC failed to induce HMGCR degradation in the UBIAD1 (G186R)-expressing stable cells (Fig 1A). In addition, the UBIAD1 (G186R) mutation had no obvious effect on SREBP-2 processing (Fig 1A). We next validated these findings using co-transfection experiments. Co-expression of HMGCR with Insig-1 conferred sterol-regulated degradation of endogenous HMGCR, as Insig-1 is a rate-limiting co-factor for multiple E3s including gp78, TRC8 and RNF145 [18–22] (Fig 1B, lanes 1–2). The WT form of mouse Ubiad1 had little effect on HMGCR degradation, whereas Ubiad1 (G184R) almost completely blocked the degradation (Fig 1B, lanes 3–6). The K89 and K248 are two ubiquitination sites of HMGCR [16], and their mutations should abolish sterol-induced degradation of HMGCR. Neither Ubiad1 (WT) nor Ubiad1 (G184R) increased the amount of HMGCR (K89R, K248R) (Fig 1B, lanes 7–12). The amount of Ubiad1 (G184R) was about 2-fold that of Ubiad1 (WT) when equivalent amounts of plasmids were used.

We next analyzed the effect of Ubiad1 on HMGCR ubiquitination. 25-HC triggered pronounced ubiquitination of immunoprecipitated HMGCR in the presence of proteasome inhibitor MG-132, which, however, was markedly reduced by WT Ubiad1 (Fig 1C, second panel, compare lane 2 and 4). The Ubiad1 (G184R) further inhibited the ubiquitination of HMGCR (Fig 1C, second panel, compare lane 4 and 6). The total amount of HMGCR from input was more abundant in Ubiad1 (G184R)-expressing cells than those expressing Ubiad1 (WT) (Fig 1C, third panel, lanes 3–6), consistent with results in Fig 1A and 1B.

We then used co-immunoprecipitation experiments to address potential interaction between HMGCR, Insig-1 and Ubiad1. The results showed that HMGCR pulled down more amounts of Ubiad1 (G184R) than Ubiad1 (WT) (Fig 1D). Meanwhile, Insig-1 association with HMGCR was reduced (Fig 1D). Together, these results suggest that SCD-associated mutant Ubiad1 competes with Insigs to bind HMGCR, thereby blocking Insig-mediated ubiquitination and degradation of HMGCR.

### UBIAD1 (G186R) enhances cholesterol synthesis

**Fig 2:**
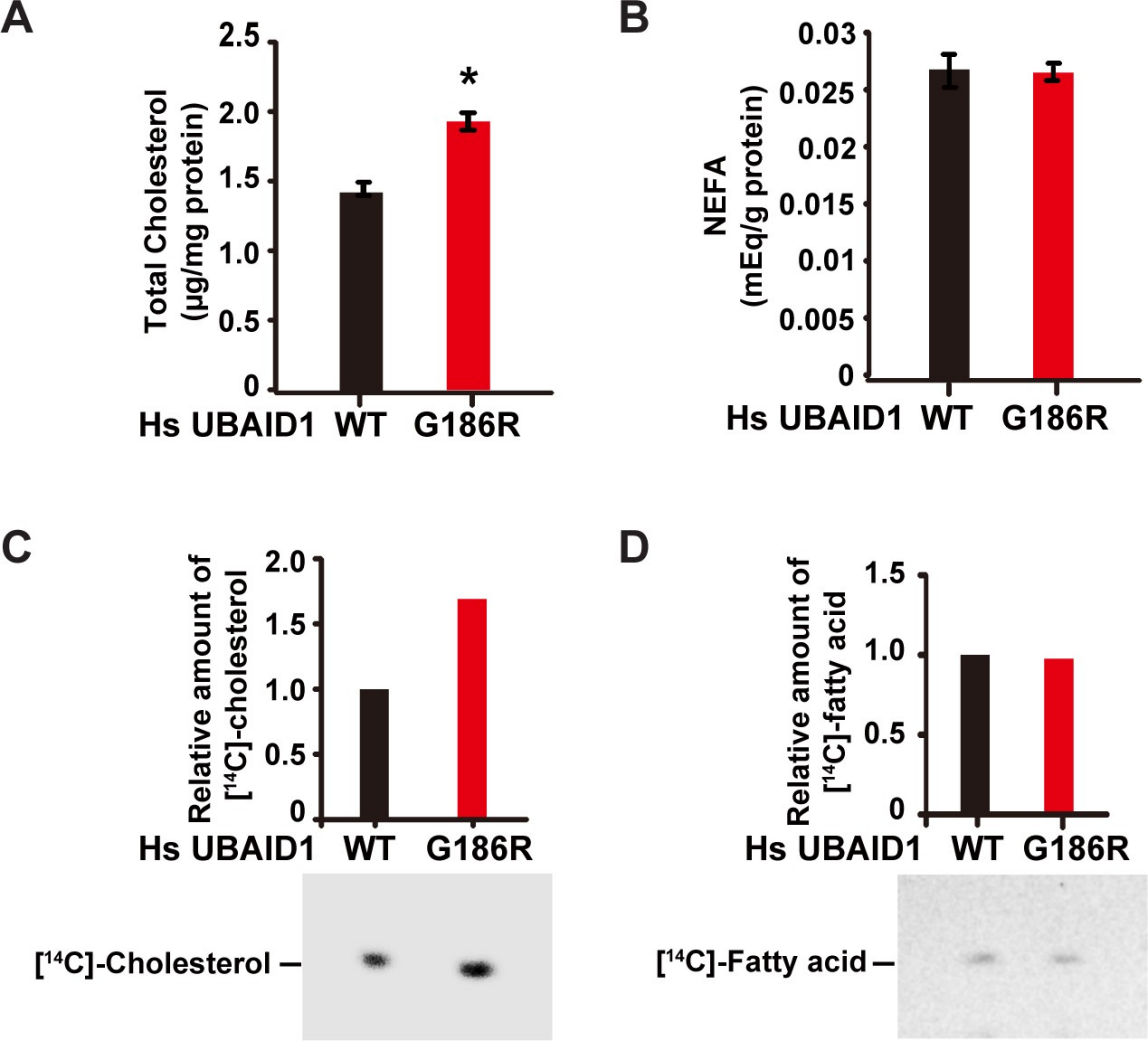
UBIAD1 (G186R) elevates cholesterol biosynthesis. (A-B) Effects of WT UBIAD1 and UBIAD1 (G186R) on the cellular levels of cholesterol (A) and non-esterified fatty acids (B). HEK-293 cells stably expressing human WT and UBIAD1 (G186R) were depleted of sterols and fatty acids in 10% delipidated-FCS, 1 μM lovastatin and 50 μM mevalonate for 16 hr. After depletion, cells were washed with PBS to remove lovastatin, and change to medium with 10% delipidated-FCS for 5 hr. Then the lipids were extracted and total cholesterol (A) and non-esterified fatty acids (NEFA) (B) were measured. The protein levels were quantified by BCA method, and used to normalize the lipid content. The data are shown as mean ± SD, n = 3. The *p* value was calculated with Student’s *t* test; *, *p* < 0.05. (C-D) Effects of WT and UBIAD1 (G186R) on *de novo* synthesis of cholesterol (C) and fatty acid (D). HEK-293 cells stably expressing with WT and G186R UBIAD1, were depleted of sterols and fatty acids in 10% delipidated-FCS, 1 μM lovastatin and 50 μM mevalonate for 16 hr. After depletion, cells were washed to remove lovastatin, and change to medium with 10% delipidated-FCS for 3 hr. Then [^14^C]-acetate (36 μCi/100-mm dish) were added and cells were treated for additional 2 hr. Cholesterol (C) and fatty acid (D) were extracted and resolved by thin-layer chromatography. Radioactive signals were visualized with phosphoimager. Signal intensities were quantified by Image-Pro Plus 6 software, and the signals in WT UBIAD1 expressing cells were defined as 1. All experiments are repeated three times and representative data are shown.

We then analyzed whether SCD-associated UBIAD1 (G186R) had any effect on cellular cholesterol level. Using the colorimetric method, we measured the amount of total cholesterol in cells stably expressing WT or G186R form of UBIAD1. UBIAD1 (G186R)-expressing cells indeed had more cholesterol than control (Fig 2A). However, no difference in the amount of nonesterified fatty acid (NEFA) was detected between these two cell lines (Fig 2B). We next sought to determine whether the G186R mutation increased *de novo* synthesis of cholesterol by using [^14^C]-acetate to label newly synthesized cholesterol and fatty acid. [^14^C]-cholesterol was markedly increased in UBIAD1 (G186R)-expressing cells (Fig 2C), whereas [^14^C]-fatty acid remained comparable between WT- and UBIAD1 (G186R)-expressing cells (Fig 2D). These results together suggest that the UBIAD1 (G186R) mutation enhances synthesis of cholesterol.

### All SCD-associated UBIAD1 mutants reduce degradation of HMGCR

**Fig 3:**
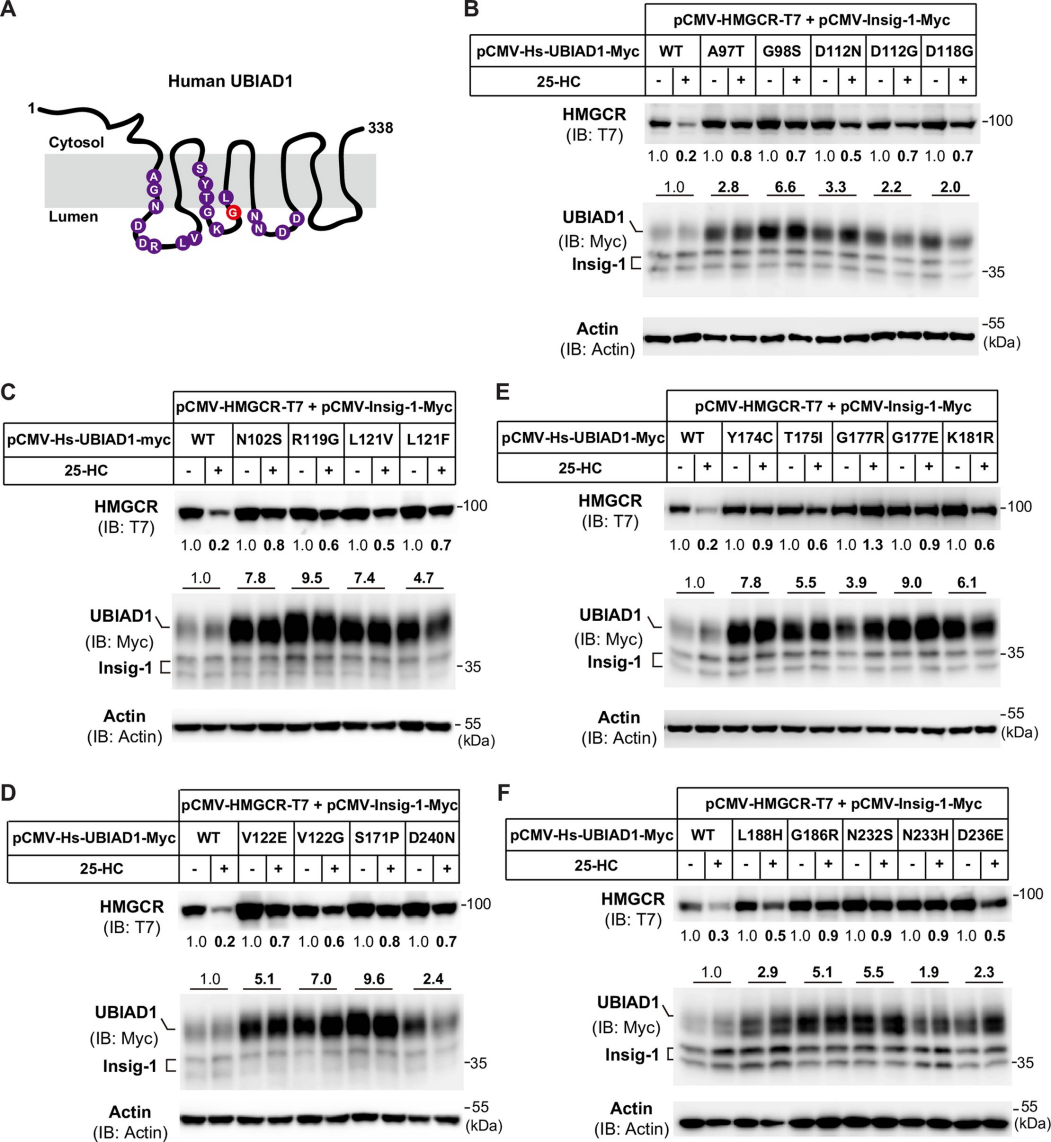
SCD-associated mutants of UBIAD1 block sterol-induced degradation of HMGCR. (A) Predicted topology of human UBIAD1 protein and SCD-associated mutations of amino acids. The G186R mutation of UBIAD1 is marked with red. There are 21 different nucleotide mutations occurred in the coding sequence that altered amino acids at 19 positions. Some residues of UBIAD1 were changed to 2 different amino acids, such as D112N, D112G, G177R, G177E, V122E and V122G. (B-F) Analysis of the effects of SCD-associated mutations of UBIAD1 on the degradation of HMGCR. CHO-K1 cells were set up in 60-mm dish and transfected with 1 μg pCMV-HMGCR-T7, 0.03 μg pCMV-Insig-1-Myc and 0.1 μg indicated WT and mutant forms of pCMV-Hs-UBIAD1-Myc per dish. 6 hr after transfection, cells were washed with PBS, and depleted of sterols in medium containing 5% LPDS, 1 μM lovastatin and 50 μM mevalonate. Following depletion for 16 hr, cells were treated with or without 1 μg/ml 25-HC and 10 mM mevalonate for 6 hr. Cells were harvested for immunoblotting with indicated antibodies. Actin was a loading control. The relative protein levels of HMGCR and UBIAD1 were quantified with Image-Pro Plus 6 software, then were normalized to the Actin loading control. The normalized intensities of HMGCR without 25-HC and mevalonate treatment were defined as 1. For UBIAD1 proteins, the normalized intensities of WT UBIAD1 bands were defined as 1, and the average intensity of two UBIAD1 bands (with or without 25-HC) are shown. The experiments are repeated three times and representative data are shown.

Human UBIAD1 contains 338 amino acids with 8 predicated transmembrane helices, and 21 SCD-associated UBIAD1 nucleotide mutations in the coding sequence that altered amino acids at 19 positions are shown in Fig 3A. Protein sequence analysis showed that amino acids that are mutated in SCD are evolutionarily conserved from fly to human (S2 Fig). As UBIAD1 (G186R) dramatically stabilized HMGCR and increased cellular cholesterol level (Fig 1, Fig 2), we next examined the effect of other SCD-associated UBIAD1 mutations on sterol-induced degradation of HMGCR. Each of the 21 SCD-associated missense mutations of UBIAD1 were co-transfected with HMGCR and Insig-1 expression plasmids individually followed by 25-HC treatment for 5 h. HMGCR protein was reduced by 80% in cells expressing the WT form of human UBIAD1 after receiving 25-HC treatment. However, 25-HC-induced HMGCR degradation was only reduced by 10% to 50% in cells expressing any of the 21 UBIAD1 mutations (Fig 3B–3F, top panel), indicating that HMGCR protein can be stabilized by SCD mutants of UBIAD1. Interestingly, the protein levels of SCD-associated UBIAD1 mutants were all substantially increased without being affected by 25-HC, even though the cells were transfected with the same amount of plasmids (100 ng per dish) (Fig 3B–3F, second panel). The protein levels of UBIAD1 harboring SCD-associated mutations were 1.9 to 9.5-fold more than WT UBIAD1 (Fig 3B–3F, second panel), while the Insig-1 protein had no changes (Fig 3B–3F, second panel). These results suggest that the SCD mutations of UBIAD1 render HMGCR proteins more resistance to sterol-induced degradation.

### UBIAD1s harboring SCD mutations are sequestered in the ER

**Fig 4:**
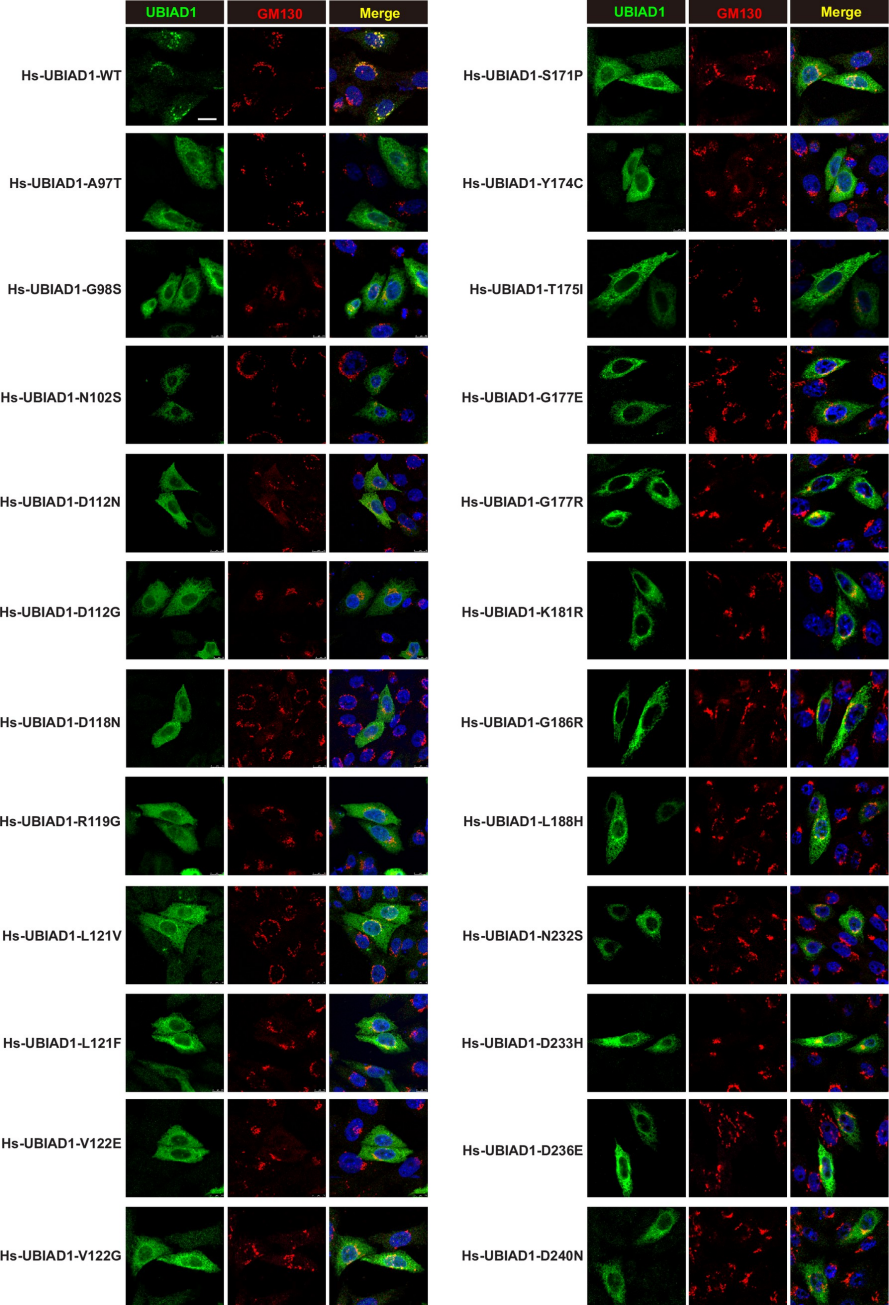
The SCD-associated UBIAD1 mutants preferentially localize on ER and WT UBIAD1 mainly localizes on Golgi. CHO-K1 cells were transfected with indicated forms of human UBIAD1 plasmids in 5% FCS medium, 1 days after transfection, cells were fixed and immunofluorescence stained with mouse monoclonal anti-Myc (against UBIAD1) antibody, rabbit polyclonal anti-GM130 (Golgi marker) antibody, and Hoechst for labeling nucleus. Scale bar, 5 μm. All experiments are repeated three times and representative data are shown.

We next examined the cellular localization of WT UBIAD1 and UBIAD1 harboring SCD mutations in CHO-K1 cells. Immunofluorescence experiments revealed that the ectopically expressed WT UBIAD1 preferentially co-localized with the Golgi marker GM130 (Fig 4). However, the SCD-associated UBIAD1 mutants had a diffused distribution and co-localized with the ER marker calnexin (Fig 4 and S3 Fig).

We further analyzed the distribution of WT and G186R UBIAD1 under different conditions. S4 Fig showed that WT UBIAD1 presented in Golgi in FCS condition and in ER in sterol-depletion condition. Nonsterol isoprenoid geranylgeraniol (GGOH), but not cholesterol or 25-HC, relocated WT UBIAD1 to Golgi (S4A and S4C Fig). However, the G186R mutant primarily located in the ER under different conditions, and seems to be trafficking-deficient (S4B and S4D Fig). We next analyzed the effects of 25-HC and GGOH on the association between HMGCR and UBIAD1. Results of S5A Fig showed that GGOH could further degrade HMGCR with 25-HC in WT Ubiad1 expressing cells, and had minimal effect in G184R mutant cells (S5A Fig). Co-immunoprecipitation results in S5B Fig showed that 25-HC stimulated HMGCR to bind more WT Ubiad1, and GGOH reduced the interaction between HMGCR and WT Ubiad1. However, the association of HMGCR and G184R mutated Ubiad1 was stronger than WT Ubiad1, and did not response to 25-HC and GGOH treatments (S5B Fig). Therefore, the SCD-associated mutations may impair ER-to-Golgi transportation of UBIAD1 and enhance UBIAD1-HMGCR interaction to prevent Insig-mediated degradation of HMGCR.

### Generation and characterization of *Ubiad1*^*G184R/+*^ mice

**Fig 5:**
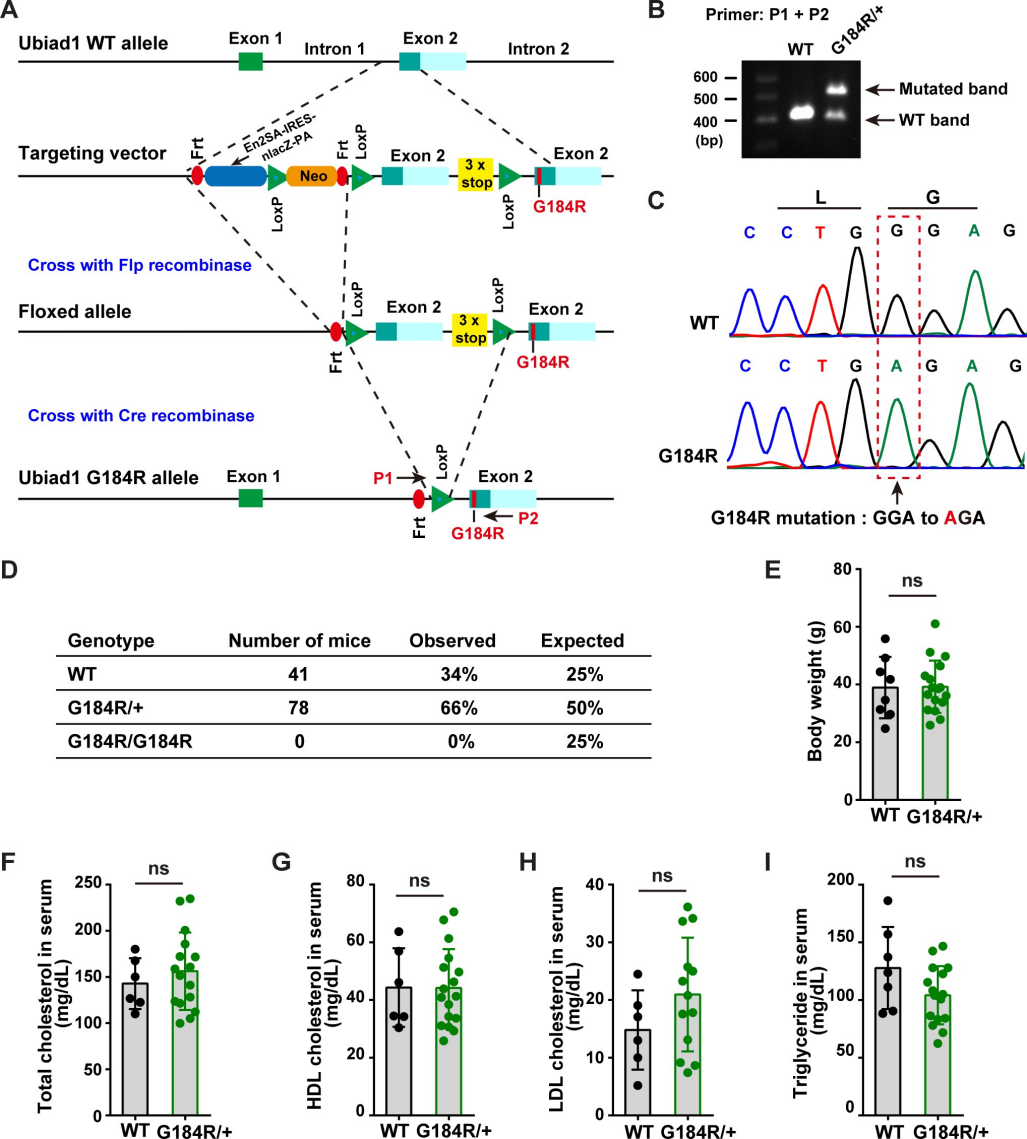
Generation and characterization of *Ubiad1*^*G184R/+*^ knock-in mice. (A) Strategy of construction *Ubiad1*^*G184R/+*^ knock-in mice. Knockout first conditional ready strategy was adopted to establish *Ubiad1*^*G184R/+*^ knock-in mice. A gene-trap cassette was inserted between intron 1 and exon 2 of *Ubaid1* allele after homologous recombination, resulting in *Ubiad1* knockout mice. Crossing with Flp recombinase-expressing strain leads to the excision of Frt-flanked cassette and the expression of WT Ubiad1 protein, as there were three stop code sequence following the inserted exon 2. Subsequently, crossing with EIIA-Cre recombinase leads to the removal of Loxp-flanked cassette and the expression of mutated G184R Ubiad1 protein. Primer 1 (P1) and primer 2 (P2) were designed to identified genotypes. Frt, Flp recognition target; Loxp, Cre recognition region; En2 SA, En2 intron 2/exon 3 splice acceptor region; IRES, internal ribosome-entry site; LacZ, β-galactosidase; PA, PolyA; Neo, neomycin. (B) Genotyping of *Ubiad1*^*+/+*^ (WT) and *Ubiad1*^*G184R/+*^ (G184R/+) mice. Genomic DNA was extracted from tail biopsies, and PCR products were amplified with P1 and P2 primers to determine the genotyping of mice. WT and mutated fragments were separated with 1% agarose gel. (C) Sequencing maps of WT and mutated band. The WT and mutated band in (B) are excised from the gel, and extracted with DNA gel extraction kit, then sequenced with P1 primer. Sequencing peak maps were visualized by Chromas software. Arrow and box indicated the G184R mutated site. (D) Breeding data of *Ubiad1*^*G184R/+*^ heterozygote intercrosses. (E-I) Male aged (102-week to 108-week old) WT (6–8 mice/group) and *Ubiad1*^*G184R/+*^ littermates (13–17 mice/group) were fed *ad libitum* with chow diet before sacrifice. Blood were harvested and subjected to the following analyses. Values are means ± SD; *p* value was calculated with Student’s *t* test; ns, no significance. (E) The body weight of WT and *Ubiad1*^*G184R/+*^ mice. (F) The total cholesterol concentration in serum from WT and *Ubiad1*^*G184R/+*^ mice. (G) The high-density lipoprotein (HDL) cholesterol level in serum from WT and *Ubiad1*^*G184R/+*^ mice. (H) The low-density lipoprotein (LDL) cholesterol level in serum from WT and *Ubiad1*^*G184R/+*^ mice. (I) The serum triglyceride level in WT and *Ubiad1*^*G184R/+*^ mice. The experiments are repeated three times and representative data are shown.

Although many missense mutations of *UBIAD1* have been found in SCD patients, the causal relationship between these two remains to be proved. To further investigate the consequence of SCD-associated mutation of UBIAD1 *in vivo*, we generated *Ubiad1* G184R (corresponding to G186R in human) knock-in mice using a knockout-first conditional ready strategy as shown in Fig 5A. The targeted allele was knocked out and conditional ready, and the mice were first crossed with Flp recombinase-expressing strain to generate the floxed allele that expressed WT Ubiad1. The mice were then crossed with EIIA-Cre recombinase transgenic mice to get the whole-body knock-in mice (Fig 5A). The WT mice had a single band of 400 bp, while the heterozygous knock-in mice (*Ubiad1*^*G184R/+*^) exhibited bands at both 400 bp and 500 bp (Fig 5B). Further sequencing of these two bands confirmed that the mutated band indeed carried the GGA-to-AGA mutation (Fig 5C).

No homozygous knock-in (*Ubiad1*^*G184R/G184R*^) mice were generated when the heterozygotes were intercrossed (Fig 5D). These results are not surprising as *Ubiad1* knockout mice defective in vitamin K_2_ synthesis are embryonic lethal as well [12]. The WT and heterozygous knock-in mice (*Ubiad1*^*G184R/+*^) were born at an expected Mendelian ratio (34% vs 66%) (Fig 5D). *Ubiad1*^*G184R/+*^ knock-in mice appeared indistinguishable from WT littermates, and both had similar body weights even at the average age of 105-week-old (Fig 5E). Given that systemic hypercholesterolemia has been reported in some but not all SCD patients [1], we next sought to evaluate cholesterol levels of WT and *Ubiad1*^*G184R/+*^ mice. The serum levels of total cholesterol (TC), high-density-lipoprotein (HDL) and low-density-lipoprotein (LDL) cholesterol in *Ubiad1*^*G184R/+*^ knock-in mice were similar to those of WT mice (Fig 5F, 5G and 5H). The serum triglyceride (TG) level of *Ubiad1*^*G184R/+*^ mice was slightly, but not significantly, decreased relative to WT mice (Fig 5I). Collectively, these results suggest that the G184R mutation of Ubiad1 does not affect systemic cholesterol and triglyceride levels.

### Accumulation of HMGCR protein in *Ubiad1*^G184R/+^ mouse tissues

**Fig 6:**
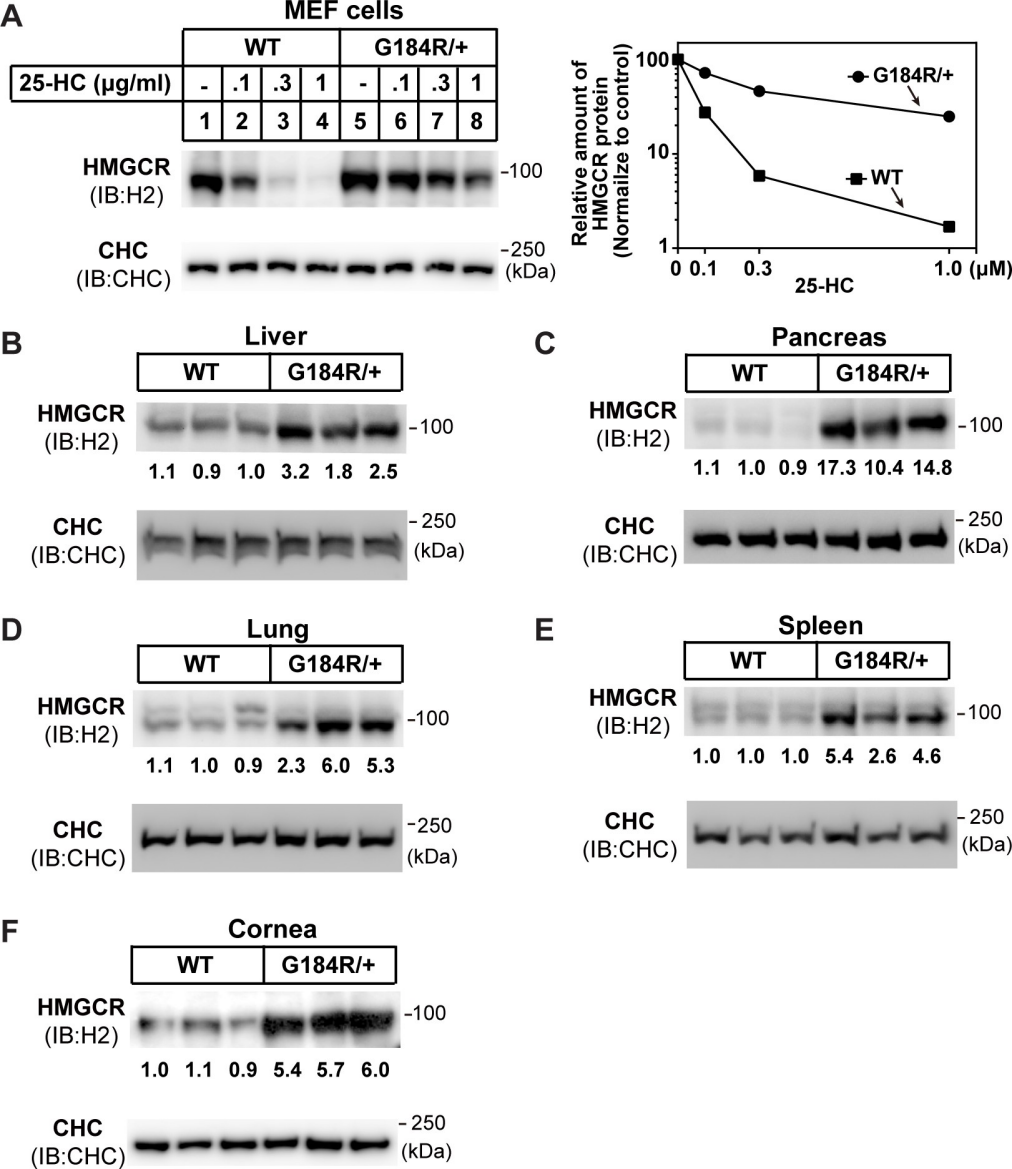
Accumulation of HMGCR protein in tissues of *Ubiad1*^*G184R/+*^ mice. (A) Regulation of HMGCR in mouse embryonic fibroblasts (MEFs) from WT and *Ubiad1*^*G184R/+*^ embryo. MEF cells were isolated and depleted of sterols in medium containing 10% LPDS, 1 μM lovastatin, 50 μM mevalonate for 16 hr, then indicated concentrations of 25-HC were added. After 5 hr incubation, cells were harvested for immunoblot analysis with rabbit polyclonal H2 (against HMGCR) and CHC. Clathrin heavy chain (CHC) was a loading control. The relative amount of HMGCR protein was quantified with Image-Pro Plus 6 software, and normalized to the CHC loading control. The normalized intensity of HMGCR band in WT MEF without 25-HC was defined as 100. (B-F) Male aged (102-week to 108-week old) WT and *Ubiad1*^*G184R/+*^ littermates were fed *ad libitum* with chow diet before sacrifice. Tissues were harvested and membrane fractions were extracted, then immunoblotted with H2 (against HMGCR) and CHC antibodies. The HMGCR protein levels of WT and *Ubiad1*^*G184R/+*^ mice in liver (B), pancreas (C), lung (D), spleen (E) and cornea (F) were immunoblotted, quantified and normalized to CHC loading control. The average amount of normalized HMGCR protein in WT group was defined as 1. The experiments are repeated three times and representative data are shown.

Since all SCD-associated point mutations including G186R impaired sterol-induced degradation of HMGCR and caused accumulation of HMGCR (Fig 1, Fig 3), we prepared mouse embryonic fibroblast (MEF) cells from *Ubiad1*^*G184R/+*^ mice and their WT littermates and analyzed HMGCR degradation. Compared with WT MEF cells, endogenous HMGCR in *Ubiad1*^*G184R/+*^ MEFs was partially resistant to ubiquitination and degradation induced by 25-HC (Fig 6A, S6 Fig), similar to the overexpression results (Fig 1A–1C). We next measured whether the G184R mutation would alter HMGCR protein levels in different mouse tissues. Strikingly, the amount of HMGCR was dramatically higher in the liver (2.5-fold), pancreas (12.1-fold), lung (4.5-fold), spleen (4.2-fold) and cornea (5.7-fold) of *Ubiad1*^*G184R*/+^ mice than WT mice (Fig 6B–6F). Together, these results indicate that Ubiad1 (G184R) protects HMGCR from degradation and increases HMGCR in various tissues.

### Accumulation of free cholesterol in the cornea of aged *Ubiad1*^*G184R/+*^ mice

**Fig 7:**
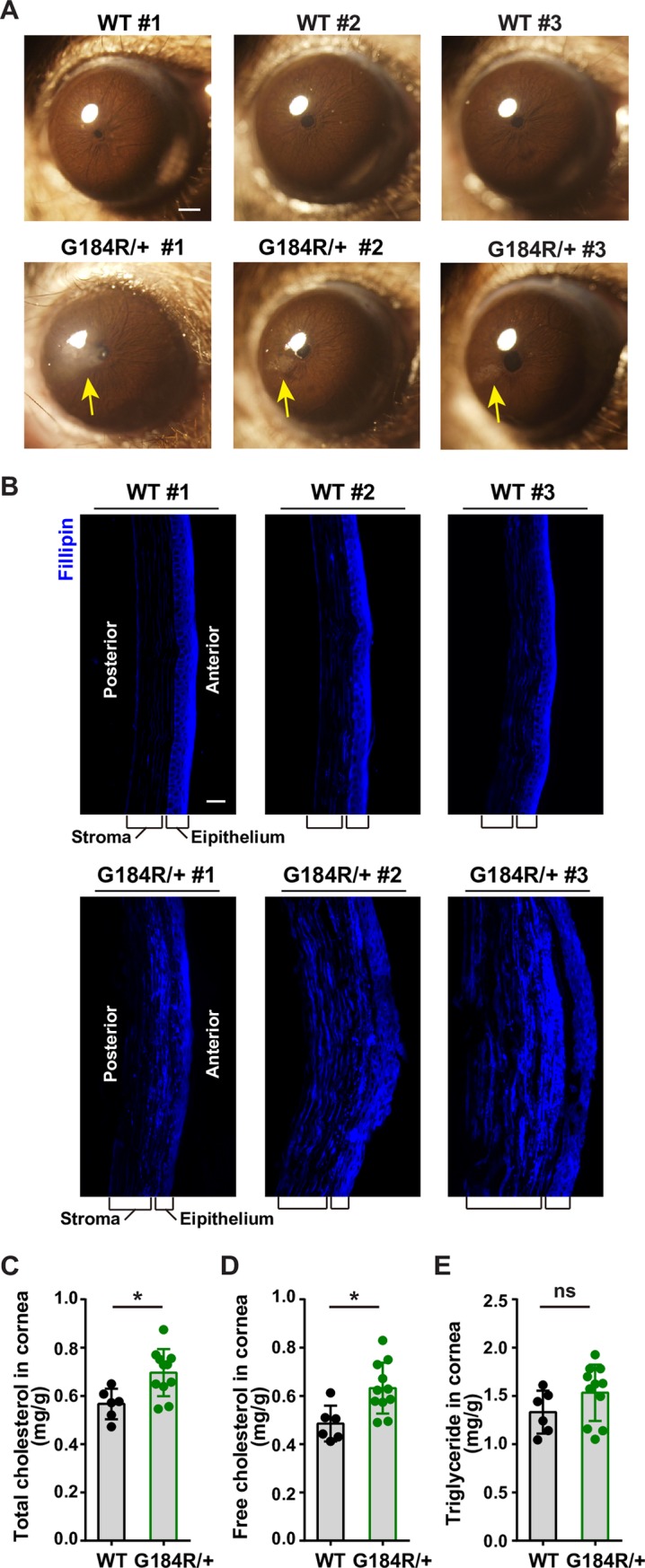
Accumulation of free cholesterol in the cornea of *Ubiad1*^*G184R/+*^ aged mice. (A) Aged *Ubiad1*^*G184R/+*^ mice show corneal opacification. Male and female 102-week to 108-week old (average: 105-week) WT (17 mice/group) and *Ubiad1*^*G184R/+*^ (33 mice/group) littermates were fed *ad libitum* chow diet. Mice were first anaesthetized with pentobarbital sodium, then corneal opacifications were observed and images were captured with Olympus stereomicroscope. Yellow arrow indicates the corneal opacification. Scale bar, 200 μm.(B) Filipin staining of corneas from WT and *Ubiad1*^*G184R/+*^ mice. Whole eyes from aged mice in (A) were dissected and sectioned at 7 μm per slide using frozen section method. Slides were stained with Filipin to visualize free cholesterol. Scale bar, 20 μm. (C-E) Male aged WT (6 mice/group) and *Ubiad1*^*G184R/+*^ (11 mice/group) mice from (A) were sacrificed and excised the cornea under stereomicroscope. The lipids in corneas were extracted and measured with corresponding colorimetric kits. Values are means ± SD; *p* value was calculated with Student’s *t* test; ns, no significance. The levels of total cholesterol (C), free cholesterol (D), and triglyceride (E) in corneas from WT and *Ubiad1*^*G184R/+*^mice. The experiments are repeated three times and representative data are shown.

Next, we examined mouse eyes using stereomicroscope. Prominent signs of corneal opacifications were found in 64% (21/33) of both male (11/17) and female (10/16) *Ubiad1*^*G184R/+*^ mice at 102-to-108 weeks of age (about 2 years, equivalent to the human age of 70) (Fig 7A, bottom panel). There was no difference between male and female mice. These corneal opacifications were haze-like and quite similar to human SCD (Fig 7A, bottom panel). As controls, none of the 17 aged WT littermates showed corneal opacification (Fig 7A, top panel). To further characterize this corneal opacification, the corneal section from these aged mice were stained with Filipin, a specific antibiotic binding to free cholesterol [32]. Free cholesterol in the WT cornea mainly localized to the epithelial cells and sporadically to the stromal cells as well (Fig 7B, top panel). However, in *Ubiad1*^*G184R/+*^ cornea the Filipin signals infiltrated throughout the anterior stroma underneath the epithelium in puncta or patches (Fig 7B, bottom panel), which correspond to the cholesterol crystals observed in the corneas of human SCD patients. Biochemical analysis showed that *Ubiad1*^*G184R/+*^ mice had higher levels of total and free cholesterol in the cornea than WT littermates, although both showed similar corneal TG levels (Fig 7C–7E). In contrast, the levels of total cholesterol, free cholesterol and TG levels in the liver, pancreas, lung and spleen were similar between WT and *Ubiad1*^*G184R/+*^ mice (S7A–S7L Fig). Collectively, these results demonstrate that the G184R mutation of Ubiad1 specifically cause free cholesterol accumulation in the anterior stroma of cornea, phenocopying human SCD.

## Discussion

**Fig 8:**
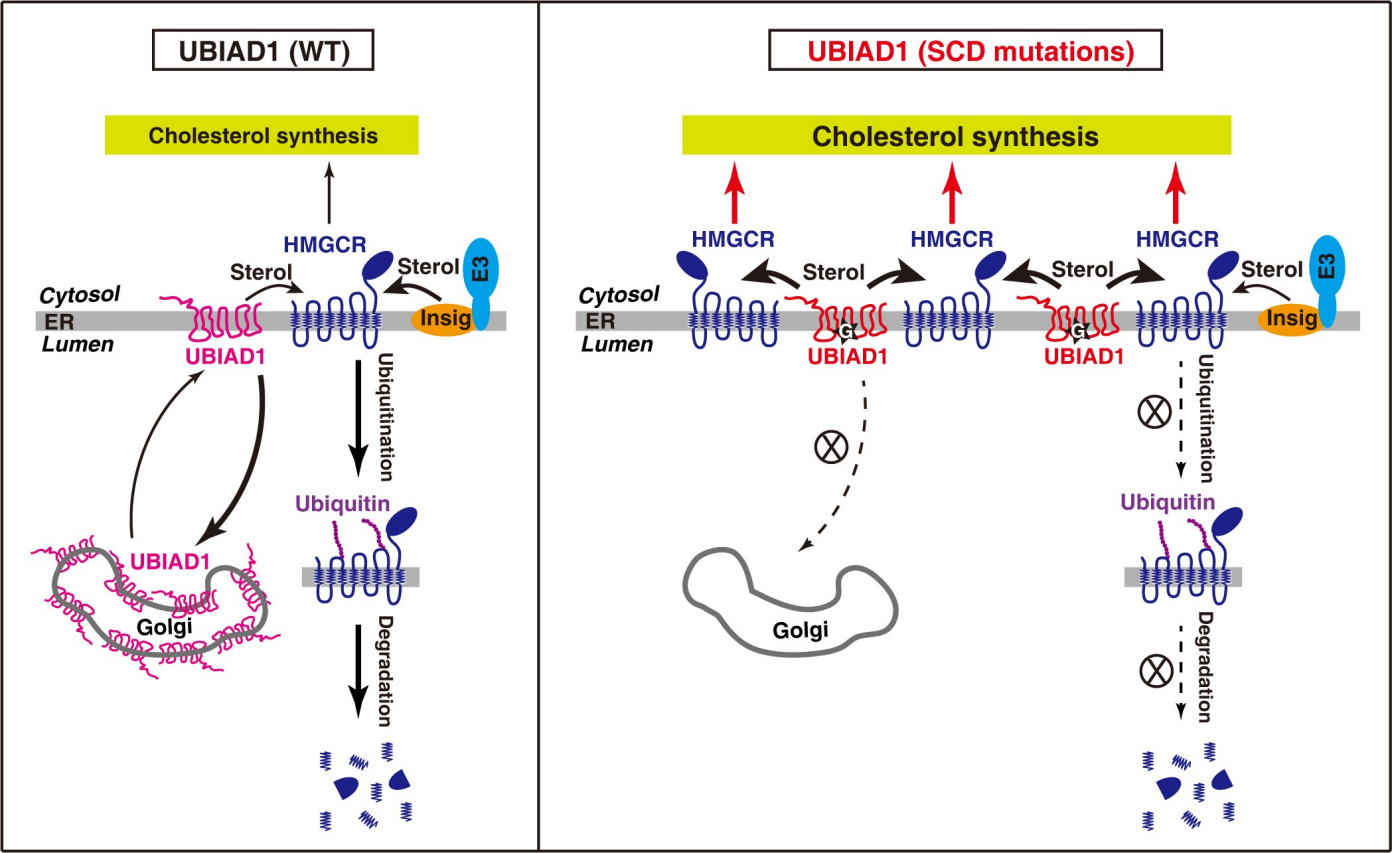
Schematic illustration of the role of UBIAD1 in sterol-accelerated degradation of HMGCR. Sterols induce ER-anchored Insig to bind HMGCR, which is ubiquitinated by Insig-associated E3 ubiquitin ligase (gp78, Trc8, or RNF145), leading to the degradation of HMGCR. In contrast to WT UBIAD1 that is efficiently transported to Golgi from ER. SCD-associated mutant UBIAD1s are sequestered in ER. They compete with Insig to bind HMGCR, inhibit the ubiquitination and degradation of HMGCR. Thus, UBIAD1 mutants increase cholesterol biosynthesis and cause cholesterol accumulation in the cornea.

Based on the above findings, we propose a working model depicting how UBIAD1 mutations cause cholesterol accumulation in the cornea (Fig 8). Under normal conditions in which the WT form of UBIAD1 mainly localizes in the Golgi, an elevation in sterols triggers HMGCR binding to Insig-1 or Insig-2, which recruits E3 ubiquitin ligases including gp78, TRC8 and RNF145 for ubiquitination and proteasomal degradation of HMGCR [18–22]. SCD-associated mutations of UBIAD1 impair its transportation from the ER to Golgi, resulting in a more stable protein in the ER that competes with Insig-1 for binding to HMGCR. As a consequence, sterol-induced ubiquitination and degradation of HMGCR is blocked. The increased cholesterol biosynthesis eventually causes cholesterol accumulation in the cornea in aged mice and humans.

The association of UBIAD1 with HMGCR was recently reported by Debose-Boyd and coworkers using HMGCR as a bait [26]. They showed that the SCD-associated mutants of UBIAD1 were sequestered in the ER and protected HMGCR from degradation, leading to the accumulation of HMGCR and cholesterol [26, 33]. They also found cholesterol accumulation in the cornea of aged *Ubiad1*^*N100S/N100S*^ mice, which is another SCD model [9]. Our results and theirs are largely consistent and both support a role of HMGCR-UBIAD1 interaction in HMGCR stabilization. However, we employed an unbiased mass spectrometry analysis of immunoprecipitated UBIAD1 and constructed a different knock-in mouse line (*Ubiad1*^*G184R/+*^). Considering that all SCD patients are heterozygotes of *UBIAD1* mutation, our *Ubiad1*^*G184R/+*^ mouse model may mimic human situation more closely. Indeed, *Ubiad1*^*G184R/+*^ mice displayed HMGCR accumulation in multiple tissues and elderly ones had free cholesterol deposition in the cornea.

Notably, corneal opacification and free cholesterol accumulation, albeit prominent in two-year-old *Ubiad1*^*G184R/+*^ heterozygote mice, were barely detected in the 3-month-old mice (data not shown). Consistently, SCD patients carrying heterozygous *UBIAD1* mutations display slow progression of corneal opacification with aging. In addition, the homozygous *Ubiad1*^*G184R/G184R*^ knock-in mice are embryonic lethal, while *Ubiad1*^*N100S/N100S*^ mice are grossly normal. Because *Ubiad1* knockout mice fail to survive through development owing to the defects in vitamin K_2_ synthesis [12], it is reasonable to speculate that the Ubiad1 G184R mutation may affect vitamin K_2_ synthesis more profoundly than Ubiad1 N100S.

Another intriguing phenomenon is that no obvious differences were detected in the serum and tissue levels of cholesterol and triglyceride between the WT and *Ubiad1*^*G184R/+*^ mice (Fig 5, Fig 7, S7 Fig), though HMGCR protein levels in all examined tissues were increased (Fig 6). Cholesterol accumulation was only detected in the cornea (Fig 7). Such phenomena are likely attributed to the unique anatomic structure of cornea, which lacks blood-vascular system and is separated from the systemic circulation [34]. Other tissues such as the liver, pancreas, lung and spleen, can intensively exchange lipids with the systemic circulation. In addition, compensatory mechanisms, such as the cholesterol esterification and efflux, may balance the cholesterol level in these tissues. According to the BioGPS expression database, the expression of acyl-CoA: cholesterol acyltransferases (ACAT)-1 and ACAT-2, which convert free cholesterol to cholesteryl ester for storage or secretion as lipoproteins [35], and ABCA1, which is an essential transporter for cholesterol efflux to HDL [36], is very low in the cornea [37]. Thus, the cornea cannot efficiently remove excess cholesterol by converting to cholesteryl ester or pumping out of the cell, leading to the accumulation of free cholesterol once HMGCR is stabilized by UBIAD1 mutations. Interestingly, familial lecithin-cholesterol acyltransferase (LCAT) deficiency and fish eye disease caused by partial loss-of-function of LCAT also exhibit evident corneal opacification due to free cholesterol accumulation [38]. These diseases highlight the importance of cholesterol homeostasis in maintaining corneal function.

Currently, corneal transplant surgery is the only way to restore vision in SCD patients [1]. It is urgent to develop other treatments in the future. As corneal overproduction of cholesterol is caused by HMGCR accumulation, it would be interesting to test whether local application of statins, the well-known inhibitors of HMGCR and are widely used for lowering cholesterol [39], in the form of eyedrops is effective for reducing corneal opacification. In addition, 2-hydroxypropyl-β-cyclodextrin (HPβCD) has an excellent ability to solubilize cholesterol and has been used to treat Niemann-Pick type C (NPC) disease caused by lysosomal cholesterol accumulation [40]. The local application of HPβCD would be another promising strategy to reduce corneal cholesterol. Our recently identified HMG499 (also named Cmpd 81) might also be used to treat SCD since HMG499 is a potent HMGCR degrader [25] and SCD is caused by HMGCR stabilization.

Collectively, we have found that the SCD-associated mutants of UBIAD1 bind and stabilize HMGCR, thereby increasing cellular cholesterol level. We have also generated and characterized a mouse model (*Ubiad1*^*G184R/+*^) for SCD disease that will be valuable for studying the underlying mechanism of SCD and developing therapeutic strategies as well.

## Methods

### Ethics statement

All procedures and care of animals were carried out in accordance with the guidelines and protocols approved by the Institutional Animal Care and Use Committee at the Wuhan University under protocol number WDSKY0201408.

### Reagents

We obtained lovastatin (PHR1285), mevalonate (41288), 25-hydroxycholesterol (H1015), MG-132 (M8699), Filipin (F9765), geranylgeraniol (G3278), protease inhibitor cocktail (P8340), N-Ethylmaleimide (E3876), and paraformaldehyde (PFA) (P6148) from Sigma; [^14^C]-acetic acid sodium salt (NEC084H001) from Perkin Elmer; and FuGENE HD transfection reagent (E2312) from Promega; G418 (345810), digitonin (300410), henylmethylsulfonyl fluoride (PMSF) (52332), leupeptin (108975), pepstatin A (516481) and ALLN (208719) from Merck; Hoechst 33342 (H1399) from Invitrogen. Lipoprotein-deficient serum (LPDS) [41] and delipidated-fetal calf serum (FCS) [42] was prepared from FCS (S1580, Biowest) by ultracentrifugation in our laboratory [25].

### Antibodies

Primary antibodies used for immunoblotting were as follows: mouse monoclonal anti-T7 (69522, Merck, 1 μg/ml), mouse monoclonal anti-Flag (F3165, Sigma-Aldrich, 1:1000), mouse monoclonal anti-Myc IgG-9E10 (CRL-1729, ATCC, 1 μg/ml), mouse monoclonal anti-ubiquitin IgG-P4D1(sc-8017, Santa Cruz Biotechnology, 1:1000), mouse monoclonal anti-hamster HMGCR IgG-A9 (CRL-1811, ATCC, 2 μg/ml), mouse monoclonal anti-clathrin heavy chain (610500, BD Biosciences, 1:1000), mouse monoclonal anti-Actin (A3853, Sigma, 1:5000), polyclonal anti-HMGCR antibody (H2) was raised against a C-terminal sequence (aa410-aa888) of human HMGCR in our laboratory [43]. Horseradish peroxidase-conjugated donkey anti-mouse (715-005-151, 1:5000) and anti-rabbit (711-005-152, 1:5000) secondary antibodies were obtained from Jackson ImmunoResearch Laboratories.

Primary antibodies used for immunofluorescence staining were as follows: rabbit polyclonal anti-GM130 (G7295, Sigma, 1:300), rabbit polyclonal to calnexin (ab22595, Abcam, 1:300). Alexa Fluor 488-labeled donkey anti-mouse IgG (A-21202, 1:500) and Alexa Fluor 555-labeled donkey anti-rabbit IgG (A-31572, 1:500) secondary antibodies were obtained from Invitrogen.

### Plasmids

The following plasmids pCMV-Insig-1-Myc, pCMV-HMGCR-T7, pCMV-HMGCR-T7-K89R/K248R, pEF1a-HA-Ubiquitin were constructed in our laboratory [25]. The coding regions of UBIAD1 was amplified from human cell cDNA or mouse liver cDNA using standard PCR and cloned into cloned into pcDNA3-C-5xMyc vector. The plasmids encoding the variants of UBIAD1 were generated by site-directed mutagenesis based on full-length human or mouse UBIAD1. All the constructs were verified by DNA sequencing. The primer sequences are listed in S2 Table.

### Cell culture

HEK-293 cells were grown in monolayer at 37°C in 5% CO_2_ in medium containing 10% FCS, Dulbecco’s modified Eagle’s medium (DMEM), 100 units/ml penicillin and 100 μg/ml streptomycin sulfate. CHO-K1 cells were grown in monolayer at 37°C in 5% CO_2_ in medium containing 5% FCS, Ham’s F-12 and DMEM (1:1), 100 units/ml penicillin and 100 μg/ml streptomycin sulfate.

### Transient transfection of CHO-K1 Cells

On day 0, CHO-K1 cells were set up for experiments at 4×10^5^ cells per 60-mm dish. On day 2, the cells were transfected with the indicated plasmids by using FuGENE HD reagent. The total amount of DNA in each transfection was adjusted to 2 μg per dish by addition of pcDNA3 mock vector.

### Immunoblot analysis

For whole cell lysate, the cells were harvested and suspended in 120 μl of RIPA buffer (50 mM Tris-HCl, pH 8.0, 150 mM NaCl, 0.1% SDS, 1.5% NP40, 0.5% deoxycholate, 2 mM MgCl_2_) containing protease inhibitors (protease inhibitor cocktail 1:500, 10 μg/ml leupeptin, 5 μg/ml pepstatin A, 25 μg/ml ALLN, 1 mM PMSF and 10 μM MG-132). Protein concentrations of the extracts were determined with Pierce BCA protein assay kit (ThermoFisher, 23225), then mixed with 4×SDS loading buffer (150 mM Tris-HCl, pH 6.8, 12% SDS, 30% glycerol, 0.02% bromophenol blue, 6% β-mercaptoethanol). Proteins were separated with SDS-PAGE, transferred to nitrocellulose filters (GE Healthcare). Immunoblots were blocked by 5% non-fat milk/TBST and probed with indicated primary and HRP-conjugated secondary antibodies. Images were captured with Amersham Imager 680 (GE Healthcare), and intensities of each band were quantified by Image-Pro Plus 6 software (Media Cybernetics).

### Membrane fraction extraction

About 50 mg tissues excised from mouse were suspended in 500 μl of buffer A (10 mM HEPES/KOH, pH 7.6, 1.5 mM MgCl_2_, 10 mM KCl, 5 mM EDTA,5 mM EGTA, 250 mM sucrose) containing protein protease inhibitors, and homogenized by Precellys 24 (Bertin). The homogenized suspensions were then passed through a #7 needle for 30 times and centrifuged at 1000 g at 4°C for 7 min. The supernatant from the 1,000 g spin was centrifuged at 1 x 10^5^ g for 30 min at 4°C. The pellets were the membrane fractions and were dissolved in 0.1 ml of SDS lysis buffer (10 mM Tris-HCl, pH6.8, 100 mM NaCl, 1% SDS, 1 mM EDTA, 1 mM EGTA).

### Generation of HEK-293/UBIAD1-WT-TAP and HEK-293/UBIAD1-G186R-TAP stable cell lines

HEK-293 cells were transfected with pCMV-Hs-UBIAD1-WT-TAP or pCMV-Hs-UBIAD1-G186R-TAP. One day later, cells were changed to medium containing 800 μg/ml G418. Fresh medium was exchanged every 2–3 days until colonies formed after about 2 weeks. Individual colonies were isolated with cloning cylinders, and the expression levels of UBIAD1 protein were verified by immunoblot.

### Tandem affinity purification coupled mass spectrometry

HEK-293/Hs-UBIAD1-WT-TAP and HEK-293/Hs-UBIAD1-G186R-TAP stable cells were set up in 100-mm dish. Cells were harvested and lysed in immunoprecipitation (IP) buffer (1% digitonin, PBS, 5 mM EDTA, 5 mM EGTA) with protein protease inhibitors. Then cells were needled 15 times and centrifuged at 13,200 rpm for 20 min. The supernatant was pre-cleared with protein A/G agarose (sc-2003, Santa Cruz Biotechnology), then immunoprecipitated with rabbit IgG coupled agarose (A2909, Sigma) at 4°C for 2 hr. Agarose-captured proteins were released with TEV Protease (P8112S, New England Biolabs) in 500 μl IP buffer at RT for 1 hr. The released proteins were immunoprecipitated with anti-Flag M2 agarose beads (A2220, Sigma) at 4°C for 2 hr, and eluted with 3x Flag peptide at 4°C for 30 min. The eluted fractions were resolved with SDS-PAGE and stained with Coomassie blue. The identifies of proteins from eluted fractions were determined by tandem mass spectrometry.

### Co-immunoprecipitation

The cells were harvested and suspended in 600 μl of IP buffer (1% digitonin, PBS, 5 mM EDTA, 5 mM EGTA, and 10 mM N-Ethylmaleimide) containing protein protease inhibitors, and passed through #7 needle 15 times. The cell lysates were centrifuged at 13,200 rpm at 4°C for 10 min, immunoprecipitated with anti-T7 antibody coupled agaroses (69026, Merck), and eluted with 2x SDS loading buffer. Whole cell lysates and pellets were subjected to SDS-PAGE and immunoblotted with indicated antibodies.

### Immunofluorescence staining

Cells were fixed with 4% PFA in PBS, permeabilized with 0.2% Triton X-100 (T8787, Sigma) for 8 minutes, blocked with 2% BSA for 1 hr and incubated with indicated primary antibodies for 1 hr at room temperature. Fluorescence-labeled secondary antibodies were used at concentration of 4 μg/ml in PBS containing 0.2% BSA for 45 minutes. Nuclei was stained with Hoechst 33342. Immunofluorescence images were captured by Leica TCS SP2 confocal microscope. Images were acquired at identical laser output, gain, and offset [44].

### Analysis of evolutionary conserved residues of UBIAD1

The protein sequences of UBIAD1 from difference species were aligned using ClustalW algorithm in MEGA X software [45]. The accession numbers of these protein sequences were used as followings: human (NP_037451.1), chimpanzee (JAA33188.1), rhesus (NP_001247708.1), tree shrew (XP_006145395.1), sheep (XP_004013772.1), horse (XP_008520311.1), elephant (XP_003413515.1), dog (XP_544571.1), rabbit (ETE69346.1), mouse (NP_082149.1), chicken (NP_001026050.1), snake (ETE69346.1), frog (NP_001016538.1), zebrafish (NP_001186655.1), sea urchin (XP_011664743.1) and fly (NP_523581.1).

### Filipin staining

Corneas were fixed by 4% PFA and sectioned with frozen cryostat (Leica CM3050 S) at 7 μm. Frozen sectioned slides were washed twice with PBS, stained with 50 μg/ml filipin in 10% FBS/PBS for 1 hr at room temperature, washed three times with PBS and mounted. Images were captured by fluorescence microscopy (Zeiss Axio Imager Z2) using a UV filter set, and the intensity of mercury lamp was turned to 10% of the maximal strength. Images were acquired at identical output, gain, and offset [46].

### Analysis of *de novo* synthesis of lipid

Cells were incubated in medium containing 10% delipidated-FCS, 1 μM lovastatin and 50 μM mevalonate for 16 hr. After depletion, cells were washed to remove lovastatin, and change to medium with 10% delipidated -FCS for 3 hr. Then [^14^C]-acetate (36 μCi/100-mm dish) were added and cells were treated for additional 2 hr. Cells were washed twice with PBS, dissolved by 0.5 ml 0.1 N NaOH, and saponified with ethanol and 75% potassium hydroxide for 2 hr. Then the nonpolar lipids (cholesterol) were extracted in petroleum ether and evaporated to dryness with N_2_. Following addition of concentrated HCl, polar lipids (fatty acids) were extracted in petroleum ether and evaporated to dryness with N_2_. The lipids were resolved by thin-layer chromatography (1.05582.0001, Merck). Radioactive signals were visualized with phosphoimager, and images were captured with Typhoon FLA 9000 (GE Healthcare).

### Generation of *Ubiad1*^*G184R/+*^ mice

The knockout first conditional ready targeting vector, containing a gene-trap cassette, was electroporated into ES cell line from C57BL/6J mice. Positive ES clones were verified by PCR and Southern blot, and three positive ES clones were injected to BALB/c blastocysts to generate chimeric mice. Male mice with high percentage chimeras were bred with female C57BL/6J mice (Shanghai Laboratory Animal Company, China) to get the heterozygous *Ubiad1* knockout mice. To get a conditional knockin allele, these heterozygous knockout mice were bred with Flp recombinase deleter transgene (Jackson Laboratory, USA) to remove the Frt-flanked cassette. Subsequently, these mice were crossed with EIIA-Cre recombinase transgenic mice (Jackson Laboratory, USA) to remove Loxp-flanked cassette, thus getting the whole tissue *Ubiad1 G184R* knockin mice. The genotypes were identified by PCR using primers P1 (GCAAGCTGTATTTTGCCTTG), and P2 (CGAAAGTGATGAGGATGACGAGGT). The male and female *Ubiad1*^*G184R*/+^ were intercrossed to get *Ubiad1*^*+*/+^ and *Ubiad1*^*G184R*/+^ mice littermates for experiments. All mice were maintained on a 12-h light/dark schedule, and fed *ad libitum* access to water and standard chow diet (Shanghai Laboratory Animal Company, China).

### Biochemistry analyses of lipids in serum and tissue

Blood were collected from anesthetized mice, and serum was prepared from blood by centrifuging at 1500 × g for 10 min. Tissues were excised and weighted, then homogenized in chloroform/methanol (2:1) with Precellys 24 (Bertin). The lipids were extracted and dried under N_2_, and dissolved in ethanol. Total cholesterol and free cholesterol levels in serum and tissues were determined with Amplex Red Cholesterol Assay Kit (A12216, Invitrogen). HDL cholesterol, LDL cholesterol and triglyceride levels in the serum and liver were measured with HDL cholesterol, LDL cholesterol and triglyceride Assay Kit (Shanghai Kehua Bio-engineering, China), respectively.

### Generation of mouse embryonic fibroblasts (MEFs)

Female *Ubiad1*^*G184R*/+^ mice were mated with male *Ubiad1*^*G184R*/+^ mice. Pregnant mice on the 13-day of conception were sacrificed by brief exposure to CO_2_. The uterine horns were excised into Petri dish and all embryos were carefully dissected. Each embryo was transferred into a new dish; the heads were used for genotyping. The rest of embryo was minced thoroughly using sharp Iris scissors, and digested with trypsin/EDTA for 20 minutes. Trypsin was neutralized, and the MEFs were collected by centrifuging. MEFs were cultured in medium with 10% FCS/DMEM, 100 units/ml penicillin and 100 μg/ml streptomycin sulfate.

### Corneal examinations

Mice were first anaesthetized with 1% pentobarbital sodium in PBS (10 μl/g), then corneal opacifications were observed and images were captured with stereoscopic microscope (Olympus SZX16). The corneas were excised from eyes and carefully removed other tissues under stereomicroscope, then used for protein and lipid analyses.

### Statistical analysis

The statistical analyses were carried out using GraphPad Prism 6 software. Data were presented as means ± SD and analyzed by unpaired two-tailed Student’s *t*-test. Statistical significance was set at *p* < 0.05.

## Supporting information

S1 Fig Tandem affinity purification coupled mass spectrometry to identify proteins associated with UBIAD1. (A) Procedure of tandem affinity purification (TAP) coupled mass spectrometry (MS). HEK-293 stably expressing Hs-UBIAD1-WT-TAP and Hs-UBIAD1-G186R-TAP was used to perform TAP-MS/MS. Cells were harvested and lysed with immunoprecipitation (IP) buffer. Lysates were first subjected to immunoprecipitation with IgG coated beads, the captured proteins were released from beads by Tobacco Etch Virus (TEV) protease cleavage. Released proteins were then immunoprecipitated with anti-Flag M2 beads, and eluted with 3×Flag peptide. The immunoprecipitated proteins were finally identified by MS/MS. Homo sapiens, Hs. (B) Coomassie blue staining of proteins interacted with UBIAD1. The immunoprecipitated proteins were separated by SDS-PAGE and stained with Coomassie blue. (C) The top three MS/MS identified proteins. Protein identities were listed with indicated spectral counts. A full list of hits identified by MS/MS is shown in S1 Table.
(TIF)

S2 Fig Amino acids of SCD-associated mutations in UBIAD1 are evolutionarily conserved ranging from fly to human. The protein sequences of UBIAD1 from 16 species were aligned using ClustalW algorithm in MEGA X software. These species include human, chimpanzee, rhesus, tree shrew, sheep, horse, elephant, dog, rabbit, mouse, chicken, snake, frog, zebrafish, sea urchin and fly. Evolutionary conserved residues are marked with asterisk, and SCD-associated mutations are marked with red.
(TIF)

S3 Fig UBIAD1s harboring SCD-associated mutations localize to ER. CHO-K1 cells were transfected with indicated human UBIAD1 plasmids and immunofluorescence stained with mouse monoclonal anti-Myc (against UBIAD1) antibody, rabbit polyclonal anti-Calnexin (ER marker) antibody, and Hoechst for labeling nucleus. Scale bar, 5 μm. The experiments are repeated three times and representative data are shown.
(TIF)

S4 Fig Geranylgeraniol regulates the transport of WT UBIAD1. (A-D) CHO-K1 cells were set up in 12-well plates with collagen-coated glass coverslips in medium with 5% FCS. On day 1, cells were transfected with 0.05 μg WT or G184R versions of pCMV-Hs-UBIAD1-Myc and 0.15 μg pcDNA3 per well. 6 hr after transfection, cells were washed twice with PBS, and depleted with 5% LPDS, 1 μM lovastatin, 50 μM mevalonate for 16 hr, then 20 μM cholesterol, 1 μg/ml 25-HC or 40 μM geranylgeraniol (GGOH) were added in the same depletion medium for 5 hr. Cells of FCS group were maintained with 5% FCS medium throughout the experiment. After 5 hr of treatment, cells were fixed and fluorescence stained with indicated antibodies as Fig 4 and S3 Fig. Scale bar, 5 μm. The experiments are repeated three times and representative data are shown.
(TIF)

S5 Fig 25-HC and geranylgeraniol regulate the interaction between UBIAD1 and HMGCR. (A) CHO-K1 cells were transfected with indicated plasmids, and depleted of sterols and isoprenoids with medium containing 5% LPDS, 1 μM lovastatin, 50 μM mevalonate for 16 hr, then 1 μg/ml 25-HC and geranylgeraniol (GGOH) were added as indicated. After 5 hr of treatment, cells were harvested and immunoblotted with indicated antibodies. (B) Cells were transfected and depleted as (A), then treated with 25-HC and GGOH for 1 hr. Cells were harvested and immunoprecipitated with anti-T7-coupled agarose beads, followed by immunoblotted with indicated antibodies. The experiments are repeated three times and representative data are shown.
(TIF)

S6 Fig Analysis the ubiquitination of HMGCR in MEFs. WT and G184R MEFs were set up in 10-cm dish with 10% FCS medium, 1 day later, cells were washed twice with PBS, depleted in medium with 10% LPDS, 1 μM lovastatin, 50 μM mevalonate for 16 hr, then 20 μM MG-132 and 1 μg/ml 25-HC were added for 2 hr. Cells were harvested and lysed, followed by immunoprecipitation with polyclonal anti-HMGCR (H2) antibody and protein A/G agarose beads. Resulting pellet and input were immunoblotted with indicated antibodies. The experiments are repeated three times and representative data are shown.
(TIF)

S7 Fig Biochemical analyses the lipid levels in tissues. (A-L) Male aged (102-week to 108-week old) WT (6–8 mice/group) and *Ubiad1*^*G184R/+*^ littermates (13–17 mice/group) were fed *ad libitum* chow diet before sacrifice. The lipids in tissues were extracted and measured with corresponding colorimetric kits. Values are means ± SD; *p* value was calculated with Student’s *t* test; ns, no significance. The levels of total cholesterol (A), free cholesterol (B), and triglyceride (C) in liver from WT and *Ubiad1*^*G184R/+*^mice. The levels of total cholesterol (D), free cholesterol (E), and triglyceride (F) in pancreas from WT and *Ubiad1*^*G184R/+*^mice. The levels of total cholesterol (G), free cholesterol (H), and triglyceride (I) in lung from WT and *Ubiad1*^*G184R/+*^mice. The levels of total cholesterol (J), free cholesterol (K), and triglyceride (L) in spleen from WT and *Ubiad1*^*G184R/+*^mice. The experiments are repeated three times and representative data are shown.
(TIF)

S1 Table Protein hits identified by MS in UBIAD1 immunoprecipitations. (XLSX)

S2 Table Primer sequences for constructing the mutated forms of UBIAD1. (DOCX)
