# Supplementary figures and images for: Schnyder corneal dystrophy-associated UBIAD1 mutations cause corneal cholesterol accumulation by stabilizing HMG-CoA reductase
Source: PLoS Genet. 2019 Jul 19;15(7):e1008289. doi: 10.1371/journal.pgen.1008289 (PMC6668851; doi:10.1371/journal.pgen.1008289)

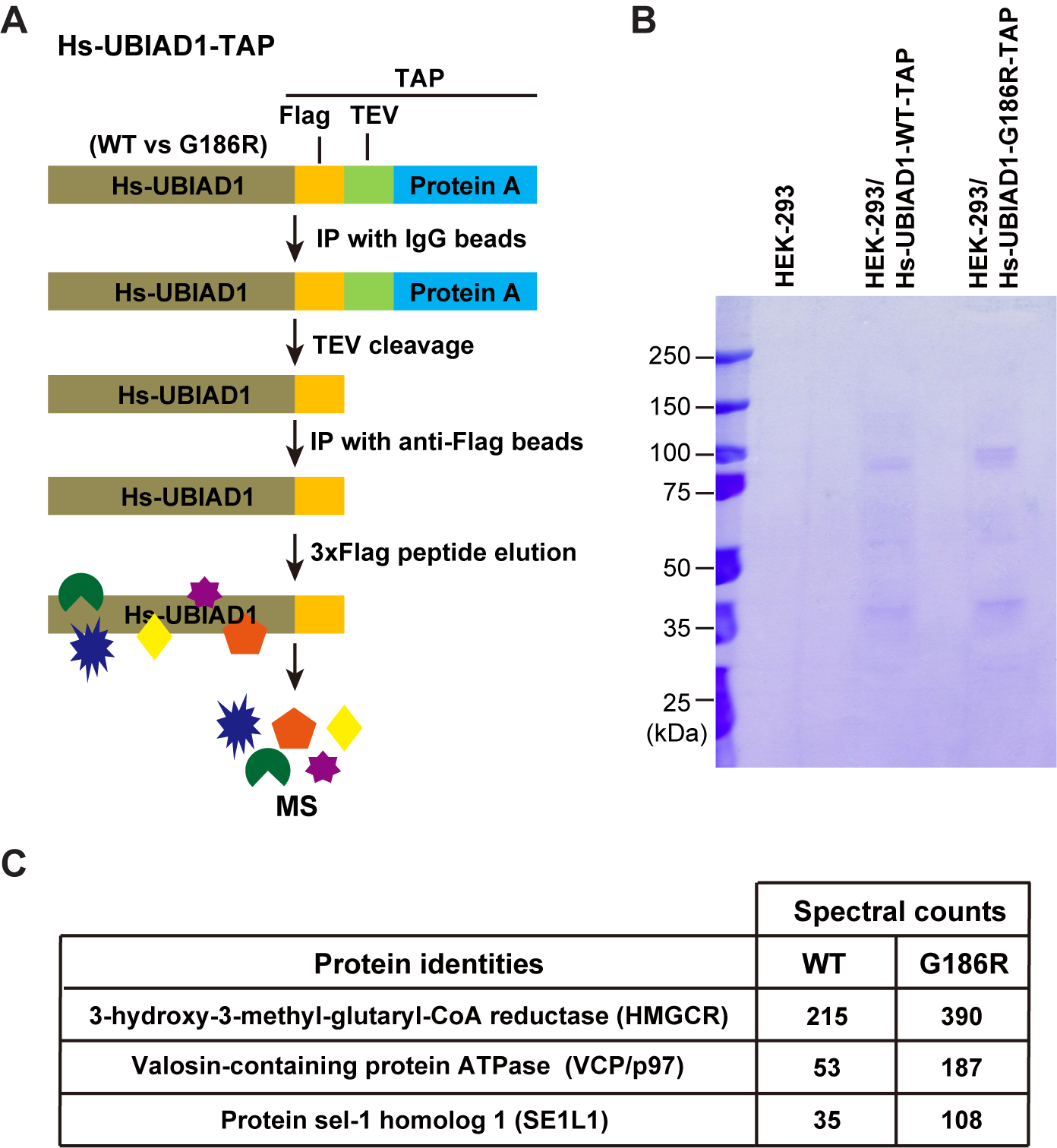

Supplement: S1 Fig — (A) Procedure of tandem affinity purification (TAP) coupled mass spectrometry (MS). HEK-293 stably expressing Hs-UBIAD1-WT-TAP and Hs-UBIAD1-G186R-TAP was used to perform TAP-MS/MS. Cells were harvested and lysed with immunoprecipitation (IP) buffer. Lysates were first subjected to immunoprecipitation with IgG coated beads, the captured proteins were released from beads by Tobacco Etch Virus (TEV) protease cleavage. Released proteins were then immunoprecipitated with anti-Flag M2 beads, and eluted with 3×Flag peptide. The immunoprecipitated proteins were finally identified by MS/MS. Homo sapiens, Hs. (B) Coomassie blue staining of proteins interacted with UBIAD1. The immunoprecipitated proteins were separated by SDS-PAGE and stained with Coomassie blue. (C) The top three MS/MS identified proteins. Protein identities were listed with indicated spectral counts. A full list of hits identified by MS/MS is shown in S1 Table. (TIF) [file pgen.1008289.s001.tif]

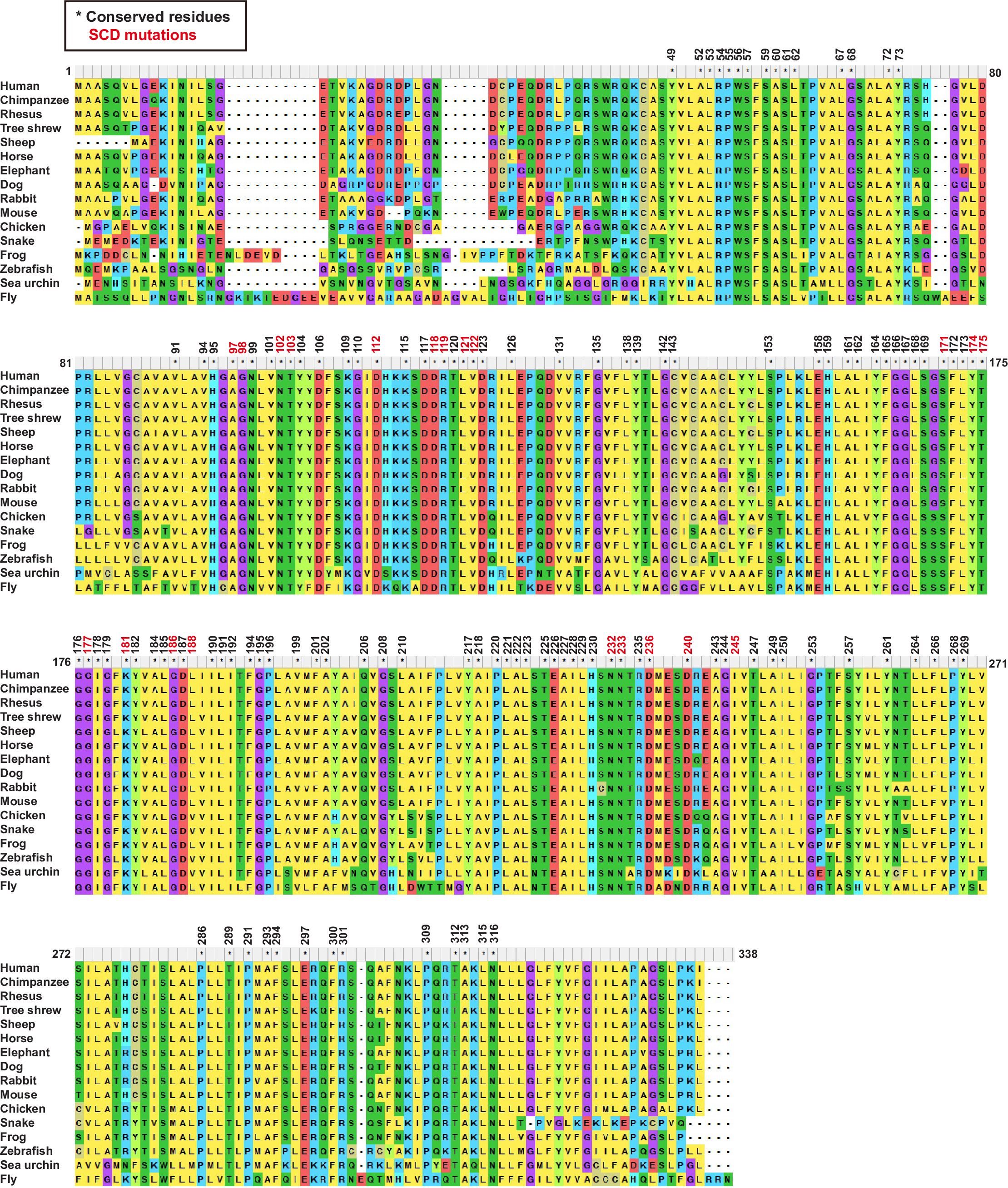

Supplement: S2 Fig — The protein sequences of UBIAD1 from 16 species were aligned using ClustalW algorithm in MEGA X software. These species include human, chimpanzee, rhesus, tree shrew, sheep, horse, elephant, dog, rabbit, mouse, chicken, snake, frog, zebrafish, sea urchin and fly. Evolutionary conserved residues are marked with asterisk, and SCD-associated mutations are marked with red. (TIF) [file pgen.1008289.s002.tif]

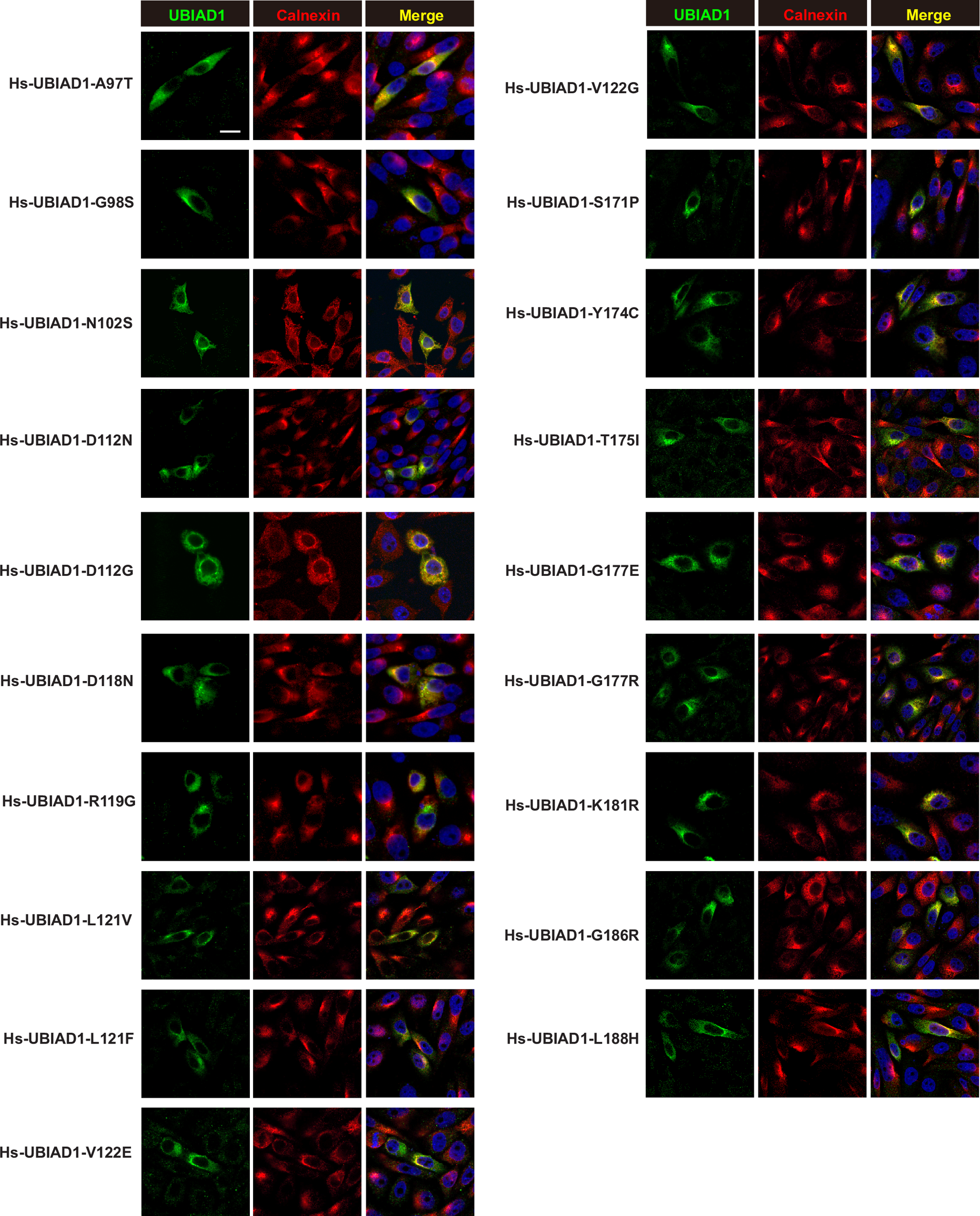

Supplement: S3 Fig — CHO-K1 cells were transfected with indicated human UBIAD1 plasmids and immunofluorescence stained with mouse monoclonal anti-Myc (against UBIAD1) antibody, rabbit polyclonal anti-Calnexin (ER marker) antibody, and Hoechst for labeling nucleus. Scale bar, 5 μm. The experiments are repeated three times and representative data are shown. (TIF) [file pgen.1008289.s003.tif]

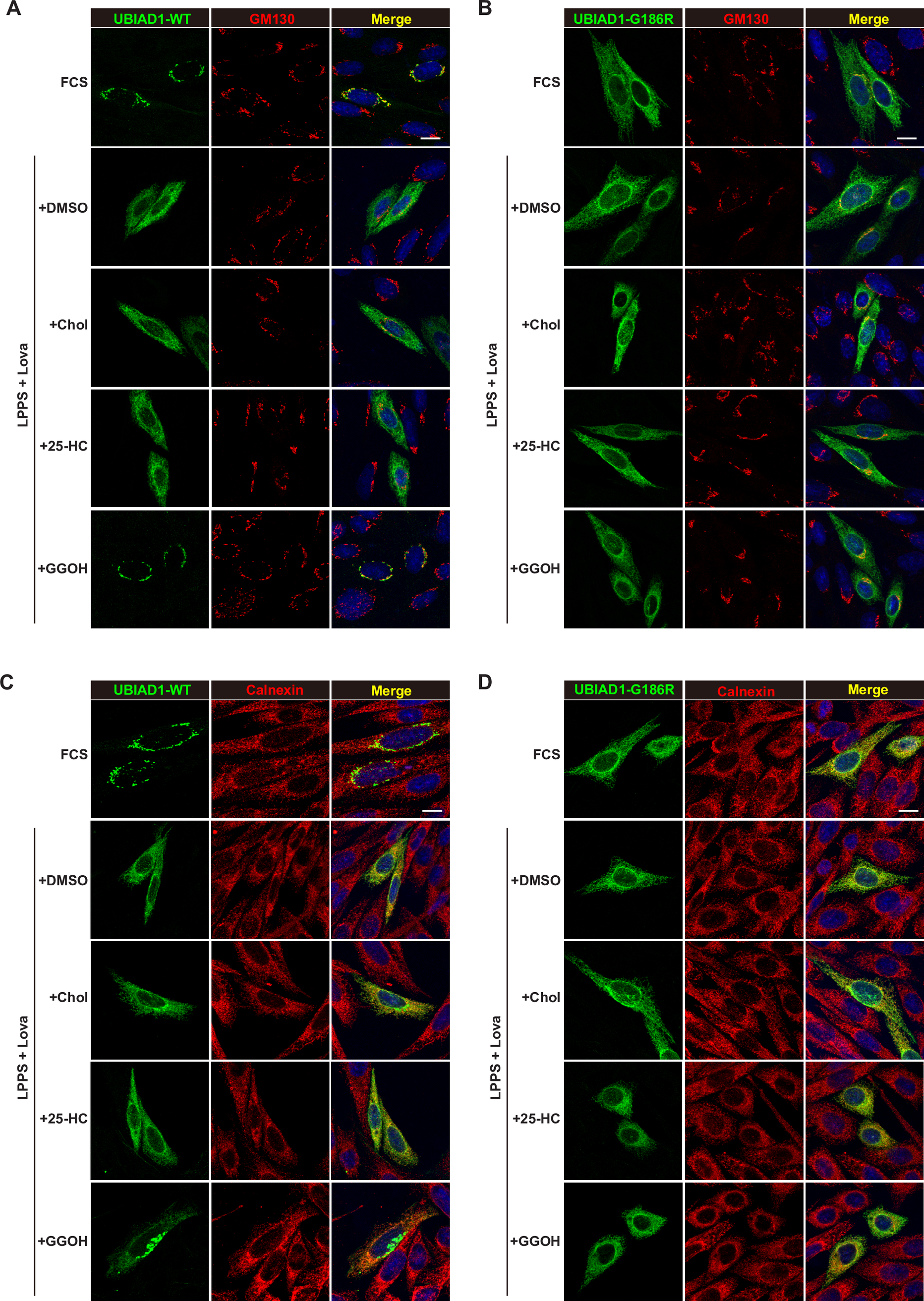

Supplement: S4 Fig — (A-D) CHO-K1 cells were set up in 12-well plates with collagen-coated glass coverslips in medium with 5% FCS. On day 1, cells were transfected with 0.05 μg WT or G184R versions of pCMV-Hs-UBIAD1-Myc and 0.15 μg pcDNA3 per well. 6 hr after transfection, cells were washed twice with PBS, and depleted with 5% LPDS, 1 μM lovastatin, 50 μM mevalonate for 16 hr, then 20 μM cholesterol, 1 μg/ml 25-HC or 40 μM geranylgeraniol (GGOH) were added in the same depletion medium for 5 hr. Cells of FCS group were maintained with 5% FCS medium throughout the experiment. After 5 hr of treatment, cells were fixed and fluorescence stained with indicated antibodies as Fig 4 and S3 Fig. Scale bar, 5 μm. The experiments are repeated three times and representative data are shown. (TIF) [file pgen.1008289.s004.tif]

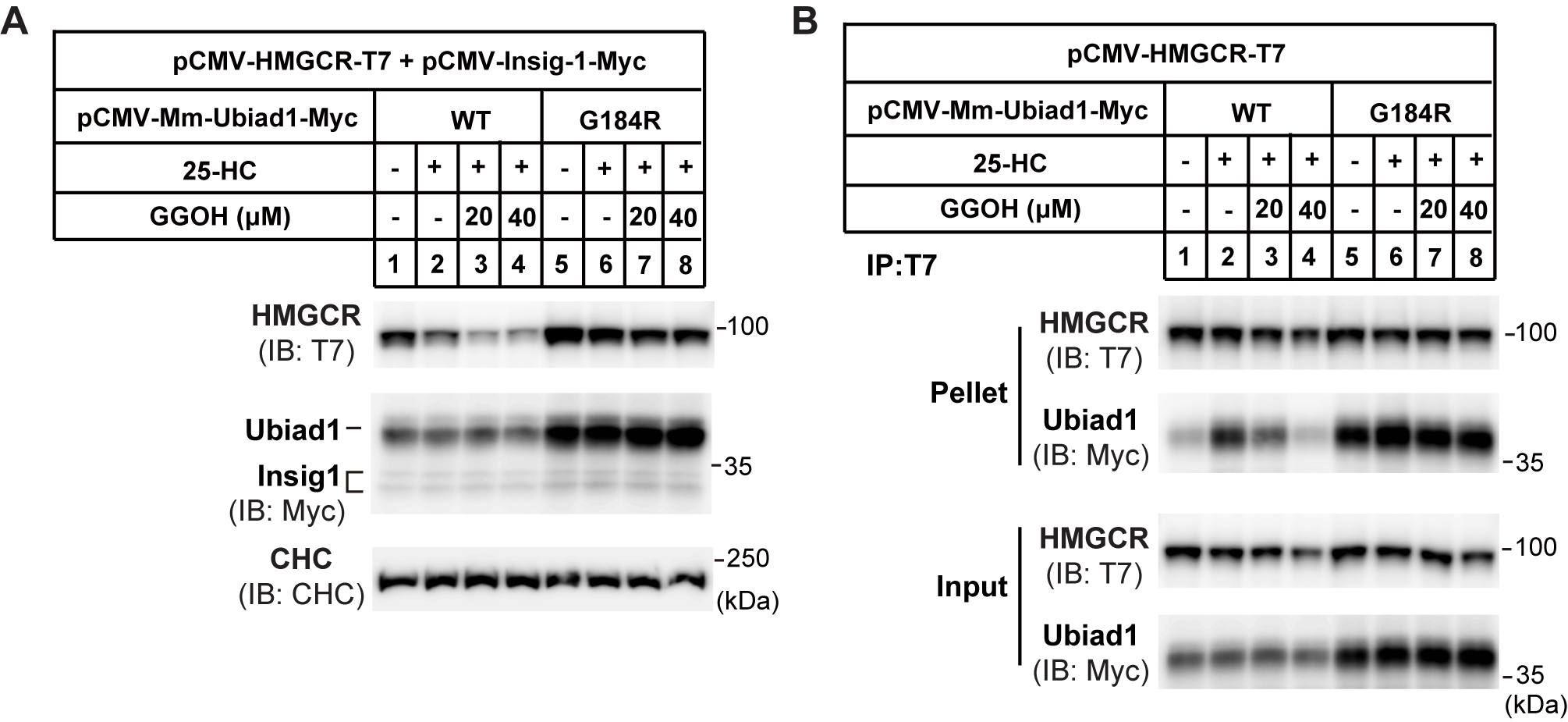

Supplement: S5 Fig — (A) CHO-K1 cells were transfected with indicated plasmids, and depleted of sterols and isoprenoids with medium containing 5% LPDS, 1 μM lovastatin, 50 μM mevalonate for 16 hr, then 1 μg/ml 25-HC and geranylgeraniol (GGOH) were added as indicated. After 5 hr of treatment, cells were harvested and immunoblotted with indicated antibodies. (B) Cells were transfected and depleted as (A), then treated with 25-HC and GGOH for 1 hr. Cells were harvested and immunoprecipitated with anti-T7-coupled agarose beads, followed by immunoblotted with indicated antibodies. The experiments are repeated three times and representative data are shown. (TIF) [file pgen.1008289.s005.tif]

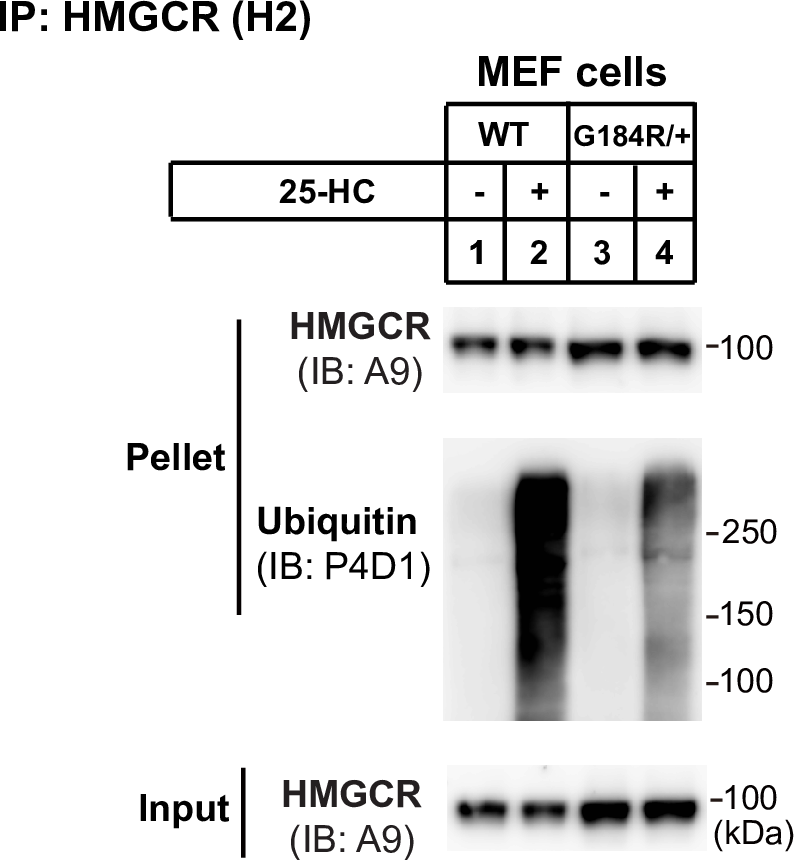

Supplement: S6 Fig — WT and G184R MEFs were set up in 10-cm dish with 10% FCS medium, 1 day later, cells were washed twice with PBS, depleted in medium with 10% LPDS, 1 μM lovastatin, 50 μM mevalonate for 16 hr, then 20 μM MG-132 and 1 μg/ml 25-HC were added for 2 hr. Cells were harvested and lysed, followed by immunoprecipitation with polyclonal anti-HMGCR (H2) antibody and protein A/G agarose beads. Resulting pellet and input were immunoblotted with indicated antibodies. The experiments are repeated three times and representative data are shown. (TIF) [file pgen.1008289.s006.tif]

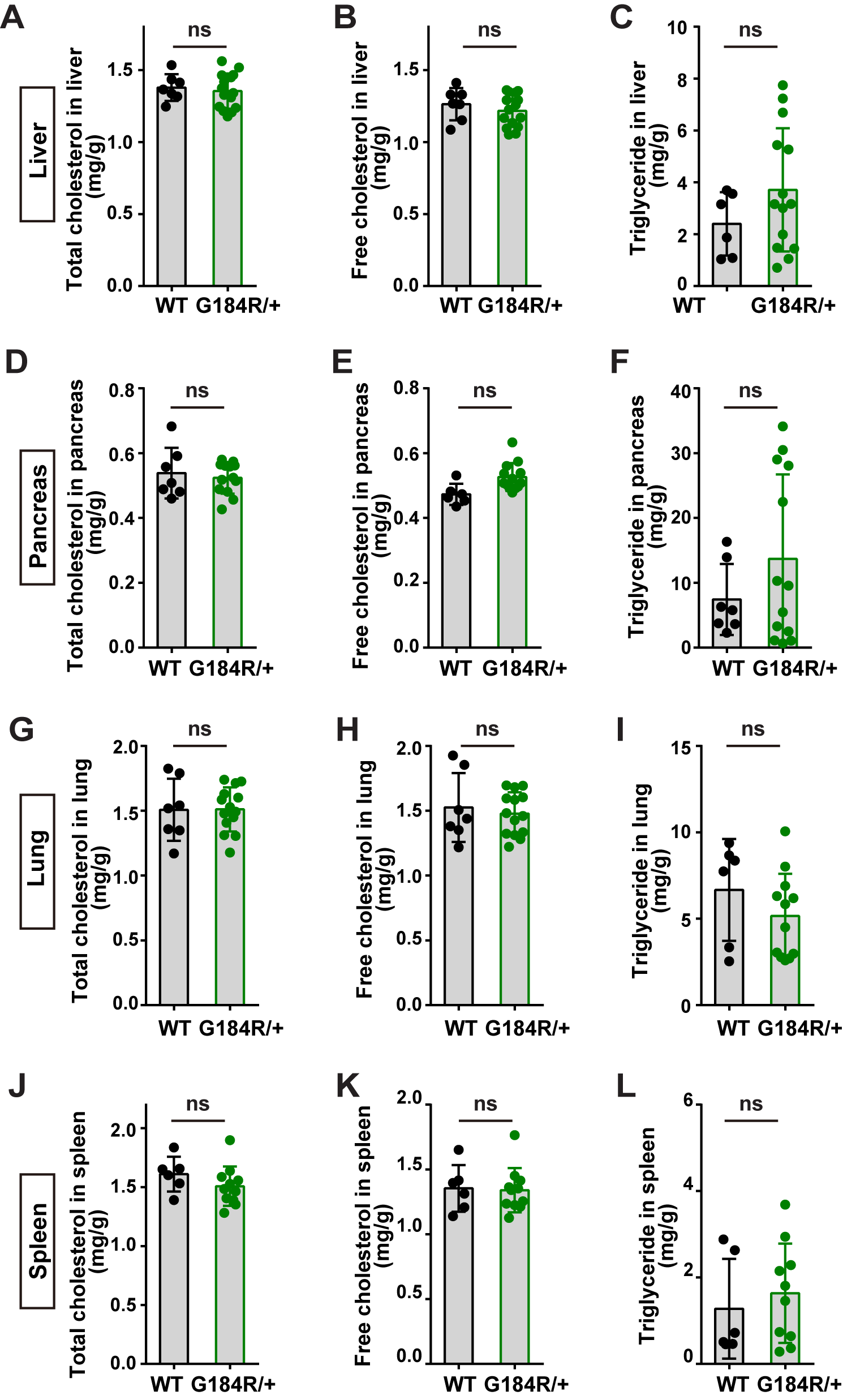

Supplement: S7 Fig — (A-L) Male aged (102-week to 108-week old) WT (6–8 mice/group) and Ubiad1G184R/+ littermates (13–17 mice/group) were fed ad libitum chow diet before sacrifice. The lipids in tissues were extracted and measured with corresponding colorimetric kits. Values are means ± SD; p value was calculated with Student’s t test; ns, no significance. The levels of total cholesterol (A), free cholesterol (B), and triglyceride (C) in liver from WT and Ubiad1G184R/+mice. The levels of total cholesterol (D), free cholesterol (E), and triglyceride (F) in pancreas from WT and Ubiad1G184R/+mice. The levels of total cholesterol (G), free cholesterol (H), and triglyceride (I) in lung from WT and Ubiad1G184R/+mice. The levels of total cholesterol (J), free cholesterol (K), and triglyceride (L) in spleen from WT and Ubiad1G184R/+mice. The experiments are repeated three times and representative data are shown. (TIF) [file pgen.1008289.s007.tif]
