# Supplementary material for: Schnyder corneal dystrophy-associated UBIAD1 mutations cause corneal cholesterol accumulation by stabilizing HMG-CoA reductase
Source: PLoS Genet. 2019 Jul 19;15(7):e1008289. doi: 10.1371/journal.pgen.1008289 (PMC6668851; doi:10.1371/journal.pgen.1008289)
Supplement: S2 Table — (DOCX) [file pgen.1008289.s009.docx]

**S2 Table. Primer sequences for constructing the mutated forms of UBIAD1.**

|  | **Primer sequences** |
| --- | --- |
| Mm-UBIAD1-WT-Forward | ATCAAGCTTTCCATGGCTGCGGTACAGGC |
| Mm-UBIAD1-WT- Reverse | ATCGCGGCCGCGAGTCTGGGCAGGCTGCCTG |
| Hs-UBIAD1-WT-Forward | ATCAAGCTTGGTACCATGGCGGCCTCTCA |
| Hs-UBIAD1-WT- Reverse | ATCGAATTCGCAATTTTGGGCAGACTGCCT |
| Hs-UBIAD1-TAP-Forward | ATCAAGCTTTCC CCATGGCGGCCTCTCA |
| Hs-UBIAD1-TAP- Reverse | ATCGCGGCCGCCAAATTTTGGGCAGACTGCCT |
| Hs-UBIAD1-A97T-Forward | TGTGCACGGGaCCGGTAATTTG |
| Hs-UBIAD1-A97T-Reverse | CAAATTACCGGTCCCGTGCACA |
| Hs-UBIAD1-G98S-Forward | GCACGGGGCCaGTAATTTGGT |
| Hs-UBIAD1-G98S-Reverse | ACCAAATTACTGGCCCCGTGC |
| Hs-UBIAD1-N102S-Forward | TAATTTGGTCTCCACTTACTAT |
| Hs-UBIAD1-N102S-Reverse | ATAGTAAGTGGAGACCAAATTA |
| Hs-UBIAD1-D112N-Forward | CAAGGGCATTaACCACAAAAAG |
| Hs-UBIAD1-D112N-Reverse | CTTTTTGTGGTTAATGCCCTTG |
| Hs-UBIAD1-D112G-Forward | CTTTTCCAAGGGCATTGGCCACAAAAAGAGTG |
| Hs-UBIAD1-D112G-Reverse | CACTCTTTTTGTGGCCAATGCCCTTGGAAAAG |
| Hs-UBIAD1-D118G-Forward | AAGAGTGATGgCAGGACACTTG |
| Hs-UBIAD1-D118G-Reverse | CAAGTGTCCTGCCATCACTCTT |
| Hs-UBIAD1-R119G-Forward | GAGTGATGACgGGACACTTGT |
| Hs-UBIAD1-R119G-Reverse | ACAAGTGTCCCGTCATCACTC |
| Hs-UBIAD1-L121V-Forward | TGACAGGACAgTTGTGGACCG |
| Hs-UBIAD1-L121V-Reverse | CGGTCCACAACTGTCCTGTCA |
| Hs-UBIAD1-L121F-Forward | TGACAGGACAtTTGTGGACCG |
| Hs-UBIAD1-L121F-Reverse | CGGTCCACAAATGTCCTGTCA |
| Hs-UBIAD1-V122E-Forward | AGGACACTTGaGGACCGAATC |
| Hs-UBIAD1-V122E-Reverse | GATTCGGTCCTCAAGTGTCCT |
| Hs-UBIAD1-V122G-Forward | AGGACACTTGgGGACCGAATC |
| Hs-UBIAD1-V122G-Reverse | GATTCGGTCCCCAAGTGTCCT |
| Hs-UBIAD1-S171P-Forward | CCTGTCTGGCcCCTTTCTCTA |
| Hs-UBIAD1-S171P-Reverse | TAGAGAAAGGGGCCAGACAGG |
| Hs-UBIAD1-Y174C-Forward | TCCTTTCTCTgCACAGGAGGA |
| Hs-UBIAD1-Y174C-Reverse | TCCTCCTGTGCAGAGAAAGGA |
| Hs-UBIAD1-T175I-Forward | CTTTCTCTACAtAGGAGGAATTG |
| Hs-UBIAD1-T175I-Reverse | CAATTCCTCCTATGTAGAGAAAG |
| Hs-UBIAD1-G177R-Forward | CTTTCTCTACACAGGACGAATTGGATTCAAG |
| Hs-UBIAD1-G177R-Reverse | CTTGAATCCAATTCGTCCTGTGTAGAGAAAG |
| Hs-UBIAD1-G177E-Forward | TTCTCTACACAGGAGAAATTGGATTCAAGTA |
| Hs-UBIAD1-G177E-Reverse | TACTTGAATCCAATTTCTCCTGTGTAGAGAA |
| Hs-UBIAD1-K181R-Forward | GAGGAATTGGATTCAGGTACGTGGCTCTGGG |
| Hs-UBIAD1-K181R-Reverse | CCCAGAGCCACGTACCTGAATCCAATTCCTC |
| Hs-UBIAD1-G186R-Forward | AAGTACGTGGCTCTGAGAGACCTCATCATCC |
| Hs-UBIAD1-G186R-Reverse | GGATGATGAGGTCTCTCAGAGCCACGTACTT |
| Hs-UBIAD1-L188H-Forward | TGGCTCTGGGAGACCACATCATCCTCATCAC |
| Hs-UBIAD1-L188H-Reverse | GTGATGAGGATGATGTGGTCTCCCAGAGCCA |
| Hs-UBIAD1-N232S-Forward | CCATTCTCCATTCCAGCAACACCAGGGACAT |
| Hs-UBIAD1-N232S-Reverse | ATGTCCCTGGTGTTGCTGGAATGGAGAATGG |
| Hs-UBIAD1-N233H-Forward | ATTCTCCATTCCAACCACACCAGGGACATGG |
| Hs-UBIAD1-N233H-Reverse | CCATGTCCCTGGTGTGGTTGGAATGGAGAAT |
| Hs-UBIAD1-D236E-Forward | CAACAACACCAGGGAGATGGAGTCCGACCGG |
| Hs-UBIAD1-D236E-Reverse | CCGGTCGGACTCCATCTCCCTGGTGTTGTTG |
| Hs-UBIAD1-D240N-Forward | AGGGACATGGAGTCCAACCGGGAGGCTGGTA |
| Hs-UBIAD1-D240N-Reverse | TACCAGCCTCCCGGTTGGACTCCATGTCCCT |

|  |  |
| --- | --- |
| Mm-UBIAD1-WT-Forward | ATCAAGCTTTCCATGGCTGCGGTACAGGC |
| Mm-UBIAD1-WT- Reverse | ATCGCGGCCGCGAGTCTGGGCAGGCTGCCTG |
| Hs-UBIAD1-WT-Forward | ATCAAGCTTGGTACCATGGCGGCCTCTCA |
| Hs-UBIAD1-WT- Reverse | ATCGAATTCGCAATTTTGGGCAGACTGCCT |
| Hs-UBIAD1-TAP-Forward | ATCAAGCTTTCC CCATGGCGGCCTCTCA |
| Hs-UBIAD1-TAP- Reverse | ATCGCGGCCGCCAAATTTTGGGCAGACTGCCT |
| Hs-UBIAD1-A97T-Forward | TGTGCACGGGaCCGGTAATTTG |
| Hs-UBIAD1-A97T-Reverse | CAAATTACCGGTCCCGTGCACA |
| Hs-UBIAD1-G98S-Forward | GCACGGGGCCaGTAATTTGGT |
| Hs-UBIAD1-G98S-Reverse | ACCAAATTACTGGCCCCGTGC |
| Hs-UBIAD1-N102S-Forward | TAATTTGGTCTCCACTTACTAT |
| Hs-UBIAD1-N102S-Reverse | ATAGTAAGTGGAGACCAAATTA |
| Hs-UBIAD1-D112N-Forward | CAAGGGCATTaACCACAAAAAG |
| Hs-UBIAD1-D112N-Reverse | CTTTTTGTGGTTAATGCCCTTG |
| Hs-UBIAD1-D112G-Forward | CTTTTCCAAGGGCATTGGCCACAAAAAGAGTG |
| Hs-UBIAD1-D112G-Reverse | CACTCTTTTTGTGGCCAATGCCCTTGGAAAAG |
| Hs-UBIAD1-D118G-Forward | AAGAGTGATGgCAGGACACTTG |
| Hs-UBIAD1-D118G-Reverse | CAAGTGTCCTGCCATCACTCTT |
| Hs-UBIAD1-R119G-Forward | GAGTGATGACgGGACACTTGT |
| Hs-UBIAD1-R119G-Reverse | ACAAGTGTCCCGTCATCACTC |
| Hs-UBIAD1-L121V-Forward | TGACAGGACAgTTGTGGACCG |
| Hs-UBIAD1-L121V-Reverse | CGGTCCACAACTGTCCTGTCA |
| Hs-UBIAD1-L121F-Forward | TGACAGGACAtTTGTGGACCG |
| Hs-UBIAD1-L121F-Reverse | CGGTCCACAAATGTCCTGTCA |
| Hs-UBIAD1-V122E-Forward | AGGACACTTGaGGACCGAATC |
| Hs-UBIAD1-V122E-Reverse | GATTCGGTCCTCAAGTGTCCT |
| Hs-UBIAD1-V122G-Forward | AGGACACTTGgGGACCGAATC |
| Hs-UBIAD1-V122G-Reverse | GATTCGGTCCCCAAGTGTCCT |
| Hs-UBIAD1-S171P-Forward | CCTGTCTGGCcCCTTTCTCTA |
| Hs-UBIAD1-S171P-Reverse | TAGAGAAAGGGGCCAGACAGG |
| Hs-UBIAD1-Y174C-Forward | TCCTTTCTCTgCACAGGAGGA |
| Hs-UBIAD1-Y174C-Reverse | TCCTCCTGTGCAGAGAAAGGA |
| Hs-UBIAD1-T175I-Forward | CTTTCTCTACAtAGGAGGAATTG |
| Hs-UBIAD1-T175I-Reverse | CAATTCCTCCTATGTAGAGAAAG |
| Hs-UBIAD1-G177R-Forward | CTTTCTCTACACAGGACGAATTGGATTCAAG |
| Hs-UBIAD1-G177R-Reverse | CTTGAATCCAATTCGTCCTGTGTAGAGAAAG |
| Hs-UBIAD1-G177E-Forward | TTCTCTACACAGGAGAAATTGGATTCAAGTA |
| Hs-UBIAD1-G177E-Reverse | TACTTGAATCCAATTTCTCCTGTGTAGAGAA |
| Hs-UBIAD1-K181R-Forward | GAGGAATTGGATTCAGGTACGTGGCTCTGGG |
| Hs-UBIAD1-K181R-Reverse | CCCAGAGCCACGTACCTGAATCCAATTCCTC |
| Hs-UBIAD1-G186R-Forward | AAGTACGTGGCTCTGAGAGACCTCATCATCC |
| Hs-UBIAD1-G186R-Reverse | GGATGATGAGGTCTCTCAGAGCCACGTACTT |
| Hs-UBIAD1-L188H-Forward | TGGCTCTGGGAGACCACATCATCCTCATCAC |
| Hs-UBIAD1-L188H-Reverse | GTGATGAGGATGATGTGGTCTCCCAGAGCCA |
| Hs-UBIAD1-N232S-Forward | CCATTCTCCATTCCAGCAACACCAGGGACAT |
| Hs-UBIAD1-N232S-Reverse | ATGTCCCTGGTGTTGCTGGAATGGAGAATGG |
| Hs-UBIAD1-N233H-Forward | ATTCTCCATTCCAACCACACCAGGGACATGG |
| Hs-UBIAD1-N233H-Reverse | CCATGTCCCTGGTGTGGTTGGAATGGAGAAT |
| Hs-UBIAD1-D236E-Forward | CAACAACACCAGGGAGATGGAGTCCGACCGG |
| Hs-UBIAD1-D236E-Reverse | CCGGTCGGACTCCATCTCCCTGGTGTTGTTG |
| Hs-UBIAD1-D240N-Forward | AGGGACATGGAGTCCAACCGGGAGGCTGGTA |
| Hs-UBIAD1-D240N-Reverse | TACCAGCCTCCCGGTTGGACTCCATGTCCCT |
